# Rational Control of Antibody Orientation for Enhancing Sensitivity and Specificity in Lateral Flow Immunoassays With Clinical Validation

**DOI:** 10.1002/adhm.71815

**Published:** 2026-10-09

**Authors:** Pin‐Hao Wang, Shunmuga Nathan Shunmuga Nainar, Bing‐Han Zhuang, Shun‐Mao Yang, Hong‐Cheu Lin, Yang‐Hsiang Chan

**Affiliations:** ^1^ Department of Applied Chemistry National Yang Ming Chiao Tung University Hsinchu Taiwan; ^2^ Department of Materials Science and Engineering National Yang Ming Chiao Tung University Hsinchu Taiwan; ^3^ Department of Surgery National Taiwan University Hospital Hsinchu Taiwan; ^4^ Center For Emergent Functional Matter Science National Yang Ming Chiao Tung University Hsinchu Taiwan; ^5^ Department of Medicinal and Applied Chemistry Kaohsiung Medical University Kaohsiung Taiwan

**Keywords:** antibody orientation, biotin‐streptavidin, boronic acid, lateral flow immunoassay, lung cancer diagnostics, multiplexed detection, polymer dots

## Abstract

Lateral flow assays (LFAs) are widely used for point‐of‐care diagnostics, but their sensitivity is often limited by inefficient antibody orientation and poor signal transduction. Herein, we report a universal strategy integrating orientation‐controlled antibody conjugation at the immunoprobe with a biotin‐streptavidin‐modified test line to enhance LFA sensitivity, specificity, and analytical reliability. Using an orientation‐controlled conjugation approach (P‐6, Scheme 1), antigen accessibility was maximized while nonspecific adsorption and false‐positive signals were suppressed. Compared with conventional randomly conjugated probes (P‐3, Scheme 1) on unmodified test lines, the combined P‐6/biotin‐streptavidin configuration achieved limits of detection of 0.0352 ng/mL for cytokeratin 19 fragment (CYFRA21‐1) and 0.0808 ng/mL for carcinoembryonic antigen (CEA), corresponding to sensitivity enhancements of approximately 14–19 fold. However, probe rankings get inverted across capture chemistries, indicating the importance of dual‐site orientation. The universality of this strategy was further validated using alpha‐fetoprotein (AFP), carbohydrate antigen 125 (CA125), carbohydrate antigen 15‐3 (CA15‐3), and prostate‐specific antigen (PSA). Furthermore, multiplexed LFAs using fluorescent polymer dots enabled simultaneous detection of CYFRA21‐1 and CEA with minimal cross‐reactivity and were validated in 28 clinical serum samples against the hospital reference method.

## Graphical abstract

Systematic investigation of antibody orientation strategies in lateral flow immunoassays reveals that a para‐substituted (aminomethyl)phenylboronic acid mediated probe (P‐6) combined with a biotin‐streptavidin test‐line modification achieves a synergistic 14‐ to 19‐fold sensitivity gain over conventional conjugation. Furthermore, multiplexed detection, universality across six biomarkers, and clinical validation in lung cancer serum demonstrate broad translational applicability.

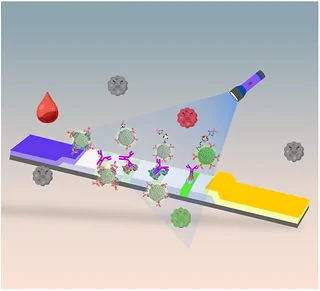

## 1 Introduction

From time to time, humanity has been exposed to deadly disease pandemics. Take, for example, the Plague of Justinian, the earliest documented pandemic [1], or the recent global crisis in 2019 (COVID‐19) [2], these events show that despite advances in medicine, rapid, low‐cost, and accurate diagnosis remains a major challenge in controlling infectious diseases. Biomarker‐based early detection could dramatically shift this balance. However, the tests most relied upon in clinical practice, “enzyme‐linked immunosorbent assay (ELISA)” and “reverse transcription polymerase chain reaction (RT‐PCR),” require centralized laboratories, costly instrumentation, and highly trained personnel [3, 4]. These demands severely restrict access in resource‐limited settings and impose unacceptable delays.

Lateral flow immunoassays (LFAs) address these barriers head‐on. They are single‐use, instrument‐free, and deliver a result as quickly as 15 min from a single drop of sample [5, 6]. The COVID‐19 pandemic demonstrated that LFAs can be produced at a billion‐unit scale and deployed across communities and low‐resource environments without specialist infrastructure. Yet that same experience exposed a critical deficiency: insufficient analytical sensitivity [7, 8]. Surprisingly, the clinical accuracy of rapid antigen LFA kits ranged from just 11% to 50% in independent evaluations [9, 10]. This limitation does not arise from manufacturing variability, but from fundamental surface chemistry governing antibody immobilization on assay interfaces.

One of the critical barriers of current LFAs is the uncontrollable orientation of antibodies [11, 12]. Notably, the antigen‐binding fragment (Fab) of an immunoglobulin G antibody must face outward from the probe or membrane surface to capture its target. However, when an antibody is adsorbed or covalently grafted at random, a substantial fraction ends up in orientations in which the Fab regions are buried, obstructed, or denatured. Moreover, the dissociation constant of an antibody‐antigen pair in free solution spans 10^−10^ to 10^−9^ mol/L. On an immobilized surface, the same pair degrades to 10^−7^ to 10^−5^ mol/L, a three‐order‐of‐magnitude loss in affinity [13]. Covalent coupling reactions, used in conventional LFA probe preparations, such as carbodiimide (EDC/NHS)‐mediated conjugation, offer only limited control over antibody orientation, often linking via undesirable amine residues near the antigen‐binding sites. As a result of this random covalent coupling, reports indicate that only around 4% of immobilized antibodies remain functionally active [14]. Similarly, the antibodies conjugated via physical adsorption, another commonly used method, retain only ∼27% of their antigenic activity [15]. Therefore, it is clear that in a conventional LFA, more than 95% of the signal‐generating material is wasted.

To remedy this with orientation‐directed immobilization chemistries, several strategies were developed on nanoprobes and test line surfaces [12, 13, 16, 17, 18, 19, 20, 21, 22]. Despite notable advances, existing orientation‐control strategies inevitably entail several trade‐offs that limit their universal implementation across all immunoassay systems. These include: i) Fc‐binding proteins, which engage the CH_2_‐CH_3_ domain of the IgG Fc region and present Fab arms away from the immunoprobe. However, this is a noncovalent, reversible, and strongly pH‐dependent interaction, with an optimal binding at pH 4.7, a condition incompatible with biological matrices [13, 17]. ii) Glycan‐targeted strategies tend to explore the N‐linked oligosaccharides of the Fc region through oxidative biotinylation or boronic acid esterification to install a capture handle with minimal impact on Fab function [18, 19, 20]. iii) Site‐specific enzymatic or chemical conjugation strategies provide excellent control over antibody orientation but typically rely on engineered antibodies, specialized reagents, or labor‐intensive workflows [21]. iv) Emerging alternatives based on antigen fragments or recombinant Fc‐capture domains offer reduced size and improved stability but remain less standardized and commercially mature [22]. Despite these individual advances, no study has simultaneously and quantitatively decoupled the orientation contributions at both interfaces of the LFA (the probe and the test line) and resolved how they combine to determine overall assay performance. Regardless, none of these orientation chemistries is new to the present work. Fc affinity capture, boronate glycan esterification, and biotin‐streptavidin coupling are all established methods. However, their behavior under identical assay conditions needs to be rigorously studied. Furthermore, it is equally important to unveil how the two assay interfaces interact.

Here, we report a dual‐site orientation framework that simultaneously engineers antibody presentation at both the nanoprobe surface and the test line capture interface. We choose semiconducting polymer dots (Pdots) as the nanocarriers, owing to their exceptional reporting behaviors in biomedical diagnostics. Their fluorescence brightness per particle exceeds that of quantum dots and organic dyes by orders of magnitude, their emission is tunable across the visible and near‐infrared spectrum, and their aqueous photostability far surpasses that of conventional fluorescent probes [23, 24, 25, 26, 27, 28]. Crucially, their surface chemistry is modular: the PSMA and DSPE‐PEG‐COOH amphiphiles used in their preparation present dense carboxylic acid groups that are amenable to a wide range of conjugation strategies without modifying the conjugated polymer core. These properties make Pdots ideal model probes for a systematic study of antibody orientation. Therefore, based on the Pdots, we construct six immunoprobe architectures (P‐1 to P‐6) that cover the full spectrum of orientation strategies: Fc‐affinity protein engagement, random covalent coupling, meta‐ and para‐substituted boronic acid Fc‐glycan targeting, and biotin‐SA capture. It is well understood that the lateral flow strip carries two orientation‐limited interfaces rather than one. First, the antigen is captured by the antibody on the nanoprobe. The probe antigen complex is then captured by the antibody on the membrane. Both events depend on how the antibody is presented in the probe. Unfortunately, every previous orientation study has primarily engineered only one of these two surfaces. Probe side orientation alone has been reported for fluorescent nanoprobes and for nanozyme labels [17, 20]. Meanwhile, capture side orientation alone has been reported for protein G and biotin‐streptavidin membranes [29, 30]. The major reviews of this field also treat orientation as a single surface problem [12, 30, 31, 32]. Unfortunately, the remaining interface is left uncontrolled in every case. This gap is of significant importance because the two interfaces are not equivalent. The probe binds free antigen in solution; however, the membrane must capture a nano‐sized particle that already carries the antigen. Here, we engineer both interfaces simultaneously. We then measure the two contributions separately and combine them. The probe contributes 6.98‐fold for CYFRA21‐1 and 5.02‐fold for CEA. Nevertheless, the test line contributes a further 2.75‐ and 2.79‐fold. Their products are 19.20 and 14.01, which match the measured overall gains. Most importantly, the two contributions are multiplicative rather than additive. We also find that probe rankings invert when the test line changes. Hence, the best probe cannot be chosen before the capture chemistry is fixed. Thus, we establish that antibody presentation geometry is a coupled two‐surface design parameter. Furthermore, each probe is characterized by four independent analytical methods, providing a degree of mechanistic rigor that has not previously been applied to this class of problem. The para‐substituted (aminomethyl)phenylboronic acid‐based probe (P‐6) emerged as the optimal probe, and its performance synergizes with a biotin‐SA‐modified test line to deliver sensitivities far exceeding those of any previously reported Pdot‐based LFA. Moreover, the platform is universal across six clinically relevant tumor biomarkers, enables multiplexed “non‐small cell lung cancer (NSCLC)” diagnostics, and is validated in clinical patient serum. In brief, this work redefines antibody orientation control as a fundamental materials engineering parameter in point‐of‐care diagnostics.

## 2 Results and Discussion

### 2.1 Design Principle of the Dual‐Site Orientation Platform

The design rationale begins with a simple observation: antigen capture in an LFA requires two sequential and independent recognition events. The first is at the Pdot probe, where a detection antibody binds the free antigen in solution. The second is at the nitrocellulose test line, where a capture antibody immobilizes the probe‐antigen sandwich. Both events depend on Fab accessibility. Optimizing only one interface (as is the standard practice) leaves the other as a performance ceiling. The present framework engineers both simultaneously and measure their contributions independently.

Six immunoprobe architectures are designed on PFCN Pdots (Scheme 1B), a visibly emissive green‐channel Pdot. Among those, P‐1 and P‐2 use protein A and protein G, respectively, as Fc‐directed coupling intermediates covalently grafted to the Pdot surface via EDC/NHS chemistry [33, 34]. Secondly, P‐3 uses conventional EDC/NHS coupling of the antibody directly to the Pdot, which serves as the benchmark control representing current practice. Thereafter, P‐4 uses 3‐aminophenylboronic acid (meta‐substituted) to form reversible boronate ester linkages with the N‐linked Fc glycans via their cis diol groups. Finally, P‐5 couples a pre‐biotinylated antibody to streptavidin‐functionalized Pdots, and P‐6 uses 4‐(aminomethyl)phenylboronic acid (para‐substituted, with an additional methylene spacer between the ring and boronic acid group) to target the same Fc glycan cis diols as P‐4, but with greater geometric flexibility. In parallel, three test line modifications (protein A, protein G, and biotin‐SA) are benchmarked against unmodified control. PFTC6FQ Pdots, a red‐channel polymer dot, are used in parallel for spectrally resolved, multiplexed CEA detection (Scheme S1) [35, 36]. Notably, CYFRA21‐1 and CEA are selected as model analytes owing to their established clinical relevance in NSCLC, the deadliest cancer worldwide [37].

**SCHEME 1:**
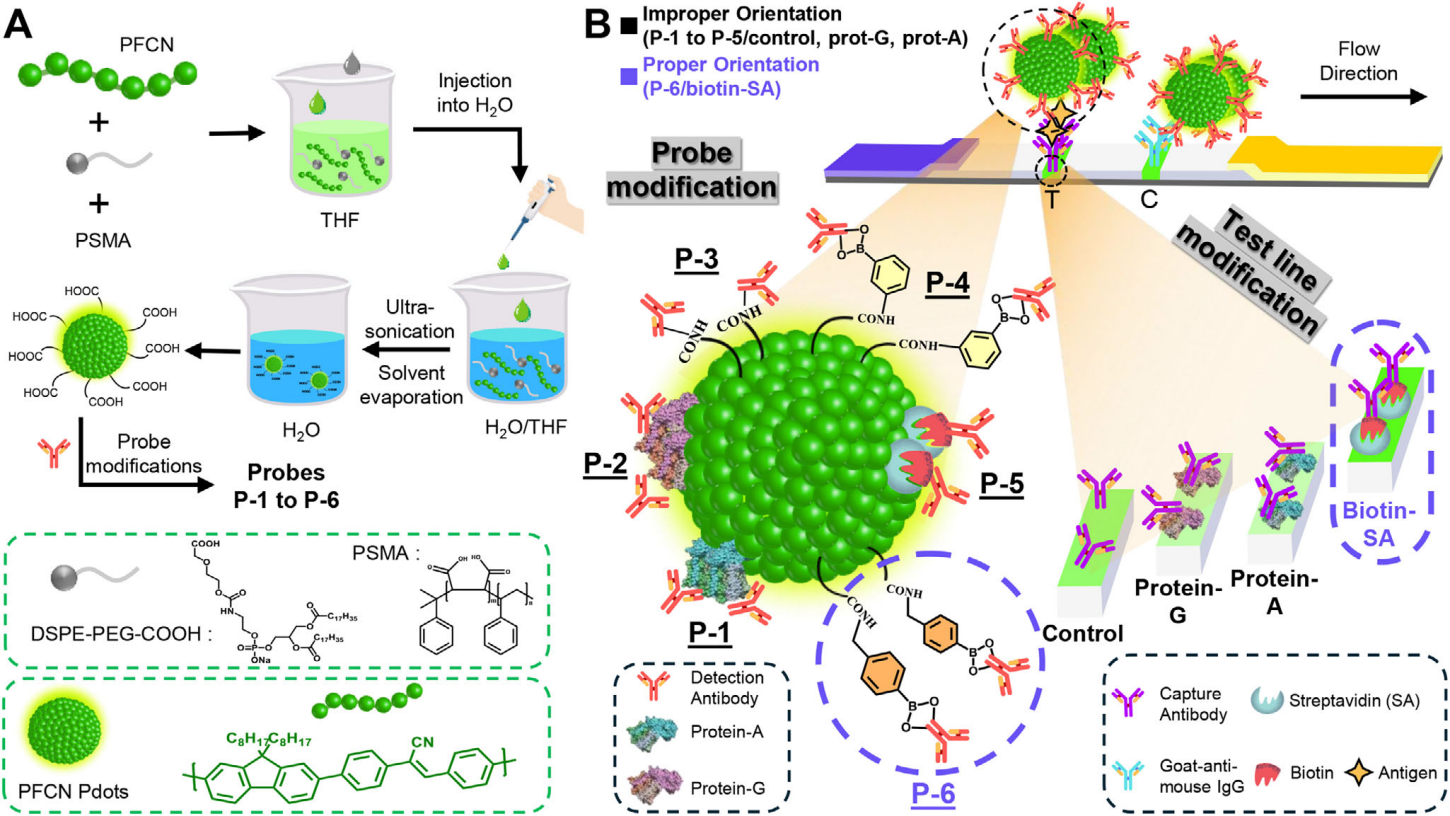
Overview of (A) the preparation of visibly emissive PFCN Pdots and (B) probe/test line modification strategies for regulating the antibody orientation in LFA.

### 2.2 Preparation and Characterization of Pdot‐Based immunoprobes

As illustrated in Scheme 1A, Pdots were prepared via nanoprecipitation and subsequently surface‐functionalized to enable antibody immobilization via distinct orientation strategies. As mentioned above, PFCN and PFTC6FQ were deliberately selected as the fluorescent polymer pair based on their complementary emission profiles and favorable spectral overlap for efficient energy transfer regulation and spectrally resolved multiplex detection (Figure 1A). Herein, PFCN was designed for CYFRA21‐1 detection, while PFTC6FQ was used for CEA detection. Both exhibit high quantum yields (PFCN: 32.5%; PFTC6FQ: 43.3%) and large molar extinction coefficients (9.22 × 10^7^ and 3.50 × 10^7^ M^−1^cm^−1^, respectively; Table 1), ensuring strong fluorescence signals at low probe concentrations. No appreciable spectral shift was observed after antibody conjugation, which indicates that the optical properties of the Pdots were largely preserved during surface modification. As displayed in Figure 1B, zeta potential data revealed the negatively charged surface of Pdots (*ζ* = ‐23.3 mV for PFCN Pdots and ‐28.9 mV for PFTC6FQ Pdots), which were largely preserved after P‐6 conjugation (*ζ* = ‐21.5 mV for PFCN/P‐6 and ‐27.4 mV for PFTC6FQ/P‐6). These values indicate that the Pdots maintain sufficient electrostatic repulsion to ensure good colloidal stability after surface modification as P‐6. We found that a slight deviation from this trend was observed in the other probe modifications, as shown in Figure S1, where *ζ* was measured to be ‐14.6, ‐13.9, ‐17.3, ‐18.1, ‐17.5, and ‐18.5 mV for PFCN/P‐1, PFCN/P‐2, PFCN/P‐3, PFTC6FQ/P‐3, PFCN/P‐4, and PFCN/P‐5, respectively. This reduction in surface charge, especially for P‐1 and P‐2, might be due to the surface carboxyl groups consumption during the immunoprobe reactions and the partial shielding of residual charges by the immobilized protein layer. Such differences in surface charge might be detrimental to subsequent detection, as reduced electrostatic repulsion can promote aggregation and impair capillary transport in LFA formats. Additionally, DLS analysis revealed slight increases in hydrodynamic diameters following antibody immobilization for P‐6 (Figure 1C–F). A similar size increase trend was observed for all other probes (Figure S1). Among the six immunoprobes, P‐6 showed the most uniform size increase with a narrow polydispersity index. In contrast, P‐1 and P‐2 displayed much broader size distributions with significant size increase, possibly due to heterogeneous Fc‐affinity binding. P‐3 showed pronounced size heterogeneity with moderate size increase, reflecting the stochastic nature of random covalent conjugation. P‐4 exhibited increased dispersity, which is attributed to the boronate‐diol interactions promoted by the rigid meta‐substituted phenylboronic acid motif. P‐5 produced relatively heterogeneous probe sizes due to the bulky and multivalent streptavidin architecture. TEM images of all probes revealed spherical Pdots with well‐defined morphology (insets in Figures 1C–F and S1A–F). The observed size and dispersity differences might influence LFA performance because excessive probe enlargement or heterogeneity can compromise capillary flow, reduce binding efficiency, and increase nonspecific interactions. Overall, these characterization results confirm that P‐6 achieves a favorable balance between effective antibody immobilization, colloidal stability, and structural uniformity, making it a strong candidate for subsequent mechanistic and analytical evaluations.

**FIGURE 1:**
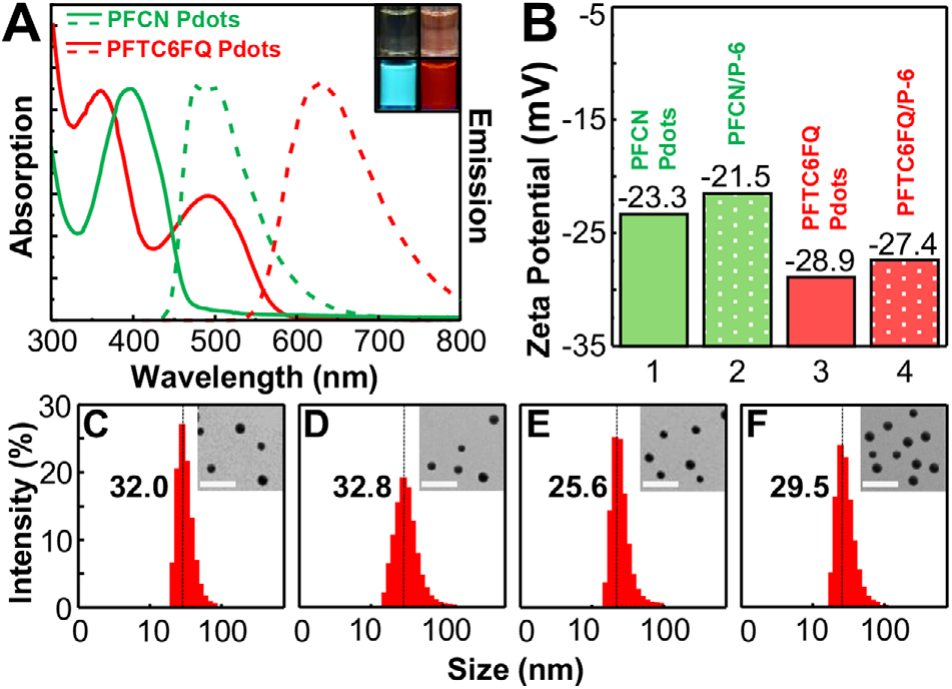
(A) UV–vis spectra (solid lines) and emission spectra (dashed lines) of PFCN and PFTC6FQ Pdots in aqueous solutions. The inset shows photographs of the two Pdot dispersions under UV illumination. (B) Zeta potentials of bare PFCN, PFCN/P‐6, bare PFTC6FQ, and PFTC6FQ/P‐6 Pdots. Hydrodynamic diameters of (C) bare PFCN, (D) PFCN/P‐6, (E) bare PFTC6FQ, and (F) PFTC6FQ/P‐6 Pdots. The insets show their corresponding TEM images. Scale bars are 100 nm. Data are mean ± standard deviation of *n* = 3 independent immunoprobe preparations. Each preparation was assayed on a separate test strip. Analysis was performed in ImageJ and OriginPro 9.1.

**TABLE 1:** Photophysical and physicochemical characteristics of Pdots.

Pdot	*λ*_abs_ (nm)	*λ*_em_ (nm)	Size (nm)	QY (%)	ε (M^−1^cm^−1^)	*τ* (ns)
PFCN	397	478	32.0	32.5	9.22 × 10^7^	0.28
PFTC6FQ	360	629	25.6	43.3	3.50 × 10^7^	1.56

Furthermore, agarose gel electrophoresis under 365 nm UV illumination (Figure S2) provides a global, probe‐wide confirmation of surface modification. All six immunoprobes, P‐1 to P‐6, display distinct electrophoretic band positions relative to bare Pdots, and the mobility shifts are consistent with the increase in hydrodynamic drag imposed by each surface modification. Nevertheless, each immunoprobe type produces a uniquely positioned and distinguishable fluorescent band, confirming that six chemically distinct surface architectures have been successfully installed on the Pdot scaffold.

The performance gap between the meta and para linkers was next examined computationally. Nevertheless, the probes are amorphous 32‐nm particles lacking symmetry, and the linkers are far too small to resolve by direct structural imaging. We therefore used density functional theory instead. Three structural observables emerge from these calculations. First, the nitrogen‐to‐boron distance is 5.01 Å in 3‐aminophenylboronic acid. The same distance is 6.56 Å in 4‐(aminomethyl)phenylboronic acid (Figure S12A‐B). Hence, the para linker projects the boron 1.55 Å further from the anchoring amide. That is an increase of about 31% in reach. Next, the meta linker populates three nearly equal rotamers with populations of 44.1%, 30.1%, and 25.8%, respectively, which span only 0.32 kcal/mol (Figure S12C). On the contrary, the para linker populates six conformers spanning 1.99 kcal/mol. Most importantly, one of these carries 71.9% of the population (Figure S12D). Next, boronate ester formation with catechol was then computed for both linkers. The binding free energies are ‐5.38 and ‐5.21 kcal/mol, respectively, for the para and meta linkers (Figure S12E). The difference of 0.17 kcal/mol is small and corresponds to only a 1.33‐fold difference in equilibrium constant per bond. This shows that both P‐4 and P‐6 can bind cis diols in nearly equal proportions, and that this is not the variable in LFA performance. Hence, the explanatory weight falls instead on reach and rotational freedom. Furthermore, relaxed dihedral scans give rotational barriers of about 6.4 and 3.1 kcal/mol for P‐4 and P‐6, respectively (Figure S12F). Thus, the barrier of the para linker is 3.26 kcal/mol lower. Nevertheless, the thermal energy at 298 K is 0.59 kcal/mol. The para barrier is therefore about 5 kBT, and the meta barrier about 11 kBT. That difference corresponds to a rotation rate about 245 times faster for the para linker. Hence, these calculations indicate that the para linker exhibits a single well‐defined preferred geometry. It also reaches its minor geometries at low energy cost. On the contrary, the meta linker has no preferred geometry and is held in whichever rotamer it first adopts. A defined yet adaptable linker must reproducibly orient an antibody population. However, a degenerate but rigid linker does not. This picture also matches the larger size dispersity measured for P‐4 by DLS (Figure S1). It matches the intermediate FRET efficiency of P‐4 as well, at 18.6%, compared with 10.2% for P‐6.

Finally, since the boronate diol association is pH dependent, the acid‐base properties were also examined [38]. Computed boronic acid pKa values are 9.60 for the P‐4 linker and 9.06 for the P‐6 linker (Figure S20A). These were obtained from an isodesmic proton exchange cycle referenced to phenylboronic acid. Both values lie above the pH of the conjugate, 7.4, and are therefore mainly neutral and trigonal during conjugation [39]. Moreover, the reactive anionic boronate is the minor species in each case. However, the 0.54‐unit difference slightly favors the para linker. We therefore tested whether it changes the amount of antibody attached. Nonetheless, antibody loading was measured by BCA assay from pH 6.5 to pH 8.5 (Figure S20B–F). Meanwhile, the antibody was offered at 1.00 mg/mL in every conjugation. It was observed that loading rises with pH for both linkers, as the boronate equilibrium predicts. Notably, it rises from about 0.60 mg/mL at pH 6.5 to about 0.85 mg/mL at pH 8.5. Moreover, the two linkers were found to differ by at most 0.030 mg/mL across all tested pH values. That is a relative difference of less than 5%, and at physiological pH 7.5, they load 0.776 and 0.777 mg/mL. The pKa difference, therefore, does not change the amount of antibody attached. The performance difference must be attributed to how well the antibody is presented. Hence, all conjugations in this work were performed in 20 mM HEPES at pH 7.4 and 37°C [40]. Since the pH was fixed, the comparison isolates the substitution pattern alone.

### 2.3 Quantitative Antibody Orientation Analysis

Four independent and complementary methods (Figure 2A–D) are used to quantify antibody orientation across all six probe configurations. Convergence across all four methods provides the most rigorous validation of orientation control reported to date in the LFA literature.

**FIGURE 2:**
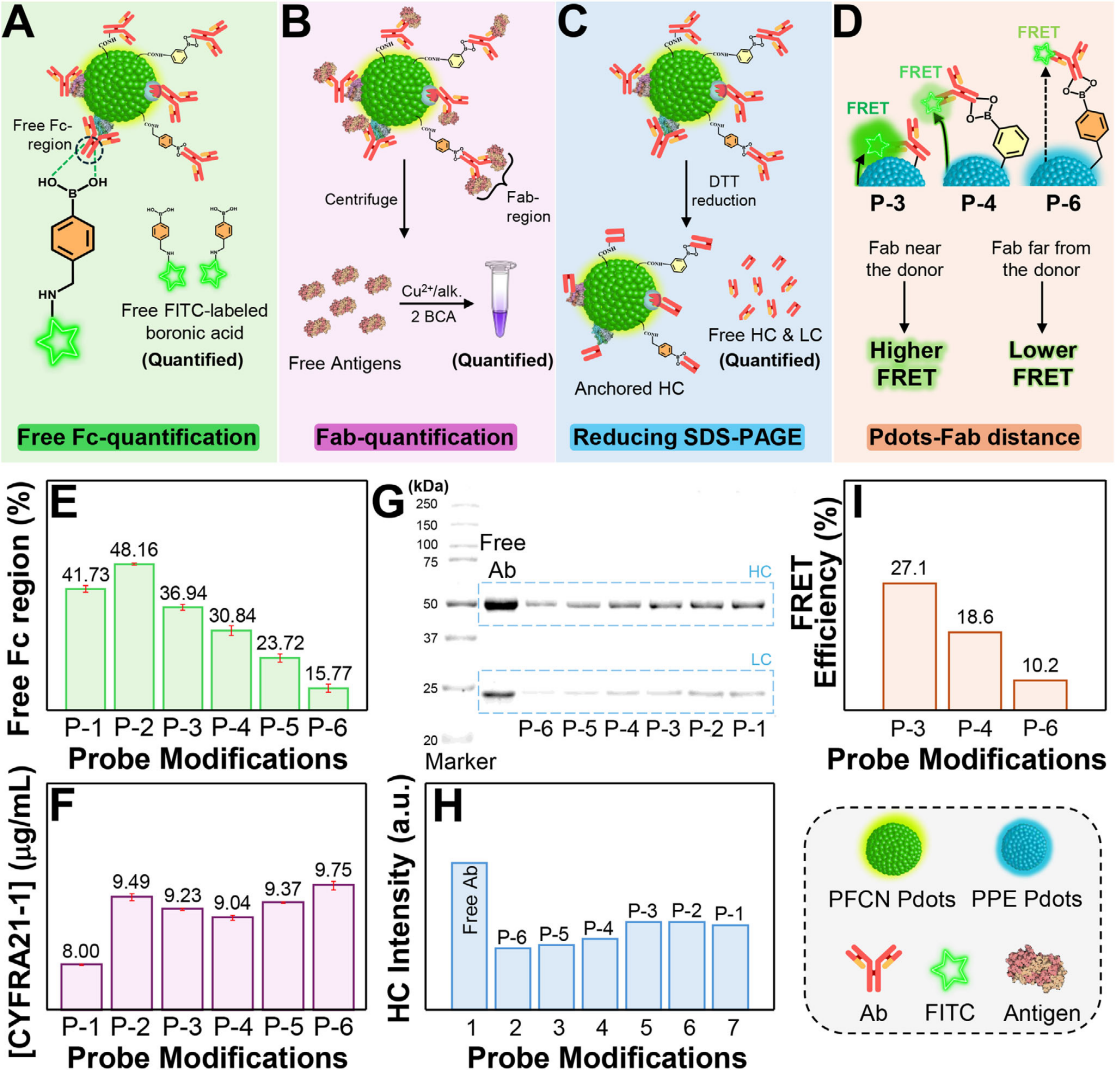
Multi‐modal validation of antibody orientation across six immunoprobe configurations. (A–D) Schematic overview of the four independent analytical methods used to quantify antibody orientation. (E) Quantification of free Fc regions derived from FITC labeling (Error bars represent the standard deviation from *n* = 3 independent measurements). (F) Antigen‐binding capacity measured by BCA assay, directly reflecting the Fab accessibility (Error bars represent the standard deviation from *n* = 3 independent measurements). (G) Reducing SDS‐PAGE gel image showing HC (∼50 kDa) and LC (∼25 kDa) bands. (H) Quantified HC band intensities normalized across immunoprobes. (I) FRET efficiencies for size‐matched immunoprobes (P‐3, P‐4, and P‐6). Data are mean ± standard deviation of *n* = 3 independent immunoprobe preparations. Each preparation was assayed on a separate test strip. Analysis was performed in ImageJ and OriginPro 9.1.

The first method uses FITC‐conjugated 4‐(aminomethyl)phenylboronic acid as a molecular probe to quantify free Fc domains (Figure S3A). This FITC‐boronic acid reporter is designed to selectively label the Fc‐associated glycan cis diols that are not engaged in surface anchoring. After incubation of each immunoprobe with the FITC reporter and centrifugal removal of unbound label, the absorbance of the supernatant was measured (Figure S3B). A standard curve (Figure S3C,D) converts absorbance to the percentage of unanchored Fc regions (see the Experimental Section). The results reveal a monotonic decrease in free Fc fraction across the probe series: 48.2% for P‐2, 41.7% for P‐1, 36.9% for P‐3, 30.8% for P‐4, 23.7% for P‐5, and only 15.8% for P‐6 (Figure 2E). This hierarchy reflects the progressive improvement in Fc‐directed surface anchoring, from affinity‐based and random immobilization strategies to glycan‐targeted and site‐specific chemistries. Nevertheless, P‐6 achieves the most effective Fc engagement, which indicates that the largest fraction of Fc domains is anchored to the P‐6 surface. The remaining Fab arms are forced into the solution phase, where they are available for antigen capture.

The second method is to quantify the antigenic activity of the immunoprobes using a bicinchoninic acid (BCA) antigen‐binding assay (Figure S4A), in which each immunoprobe is initially incubated with a fixed concentration of CYFRA21‐1 antigen (10 µg/mL). The unbound antigen in the supernatant after incubation (Figure S4B) is quantified by BCA colorimetry using a standard calibration curve (Figure S4C,D) and converted to the corresponding amount captured by the precipitated immunoprobe. Thus, higher antigen in the precipitate indicates greater antigen capture, providing a direct measure of the effective accessibility of the Fab. Again, P‐6 achieves the highest antigen retention at 9.75 µg/mL (Figure 2F), captured out of 10 µg/mL tested. P‐1 retains the least at 8.00 µg/mL. Notably, the BCA and FITC‐Fc labeling assays both place P‐6 first, although they order the intermediate probes differently. Together, they confirm that the probe with the most Fc engagement (P‐6) also achieves the greatest Fab‐mediated antigen capture. This physical logic underpins the observed LFA performance hierarchy.

The third and most direct method is reducing SDS‐PAGE with Coomassie Blue staining (Figure 2G). The initial DTT treatment cleaves disulfide bonds in surface‐conjugated antibodies, which releases heavy chains (HC, ∼50 kDa) that are unanchored from the immunoprobes, and light chains (LC, ∼25 kDa) for electrophoretic separation. The key principle is as follows. If the Fc region is firmly anchored to the Pdot surface via boronic acid bonds, the HC remains tethered to the nanoparticle after DTT reduction. It does not enter the gel, resulting in a weak or absent heavy‐chain band. On the contrary, if the Fc is free, the HC migrates normally and produces a strong band. P‐6 shows the most attenuated heavy chain band among the six probes, with HC intensity substantially lower than that of free antibody and the other five immunoprobes. A bar chart of normalized HC band intensities across all probes (Figure 2H) confirms P‐6 as the most effectively Fc‐anchored probe.

The fourth and final method is the Förster resonance energy transfer (FRET)‐based lifetime analysis. Here, poly(*p*‐phenylene ethynylene) (PPE) Pdots serve as the energy donor, and a FITC‐labeled CYFRA21‐1 antibody serves as the acceptor (Figure S5A,B) [41]. Under low degree‐of‐labeling conditions, FITC preferentially acylates solvent‐exposed lysines enriched in the Fab domains of IgG [21]. This makes FITC a Fab‐selective positional reporter. Properly oriented antibodies, with the Fc anchored to the Pdot surface, have their FITC‐labeled Fab regions maximally displaced from the donor core, thereby reducing FRET efficiency. Whereas the poorly oriented antibodies have Fab regions near the Pdot surface, increasing FRET. Notably, the FRET efficiency is physically governed by the Forster equation *E* = 1/(1 + (*r*/*R*_0_) [6]), where *r* is the donor‐acceptor distance, and *R*_0_ is the Forster radius. For the PPE Pdot‐FITC pair, *R*_0_ is calculated as 10.53 nm (see the Experimental Section). FRET is only informative when *r* is comparable to *R*_0_. This imposes a critical size constraint on the analysis. P‐1 and P‐2, with hydrodynamic radii of approximately 70 and 105 nm, place the FITC‐Fab acceptors far beyond the FRET detection range regardless of orientation. Similarly, P‐5, with its extended biotin PEG4‐amine spacer, has a Fab‐to‐surface distance that exceeds the FRET‐sensitive window. For these probes, weak quenching would reflect probe size rather than antibody orientation. FRET analysis is therefore restricted to the size‐matched subset: P‐3, P‐4, and P‐6 (Figure S5C). For this trio, FRET efficiencies track orientation precisely (Figure S5D–G), where P‐3 shows 27.1% FRET efficiency, consistent with random conjugation placing significant Fab fractions near the Pdot surface. P‐4 shows intermediate FRET at 18.6%, reflecting partial Fc‐directed orientation. P‐6 achieves the lowest FRET efficiency at 10.2%, corresponding to the greatest mean Fab‐to‐surface distance and the most uniform Fc‐directed orientation (Figure 2I). Time‐resolved fluorescence lifetime measurements corroborate these trends independently. Donor lifetimes are calculated as 0.43 ns for P‐3, 0.48 ns for P‐4, and 0.53 ns for P‐6, compared to 0.59 ns for bare PPE Pdots with no quenching. P‐6 approaches the bare Pdot lifetime most closely, confirming minimal FRET and maximum antibody extension from the surface. This ranking matches the FITC‐Fc and SDS‐PAGE results, and all four methods place P‐6 first.

The convergence of these four independent analytical methods, spanning fluorescence labeling, colorimetric protein quantification, electrophoretic separation, and energy transfer spectroscopy, constitutes the most comprehensive orientation validation reported for LFA immunoprobes, which confirms P‐6 as the optimal probe configuration.

However, the six probes differ in more than the antibody attachment site. It should be noted that the probe size, surface charge, and linker chemistry also change with the conjugation route. Any of these could, in principle, drive the observed performance difference. Thus, a further control probe, P‐3+, was prepared to separate these variables (Figure S11). To do so, we made thiol‐terminated PFCN Pdots using poly(styrene‐co‐thiol) (PSSH) with the usual amphiphiles. Next, the antibody was activated with succinimidyl 4‐(N‐maleimidomethyl)cyclohexane‐1‐carboxylate (SMCC) and then coupled to the Pdot thiols. Notably, similar to EDC/NHS‐based covalent coupling, SMCC acylates lysine residues distributed across the entire antibody surface. The antibody on P‐3+ is therefore randomly oriented by design. Apart from the attachment site, P‐3+ and P‐6 match in every descriptor that can be measured directly. DLS analysis revealed diameters of 32.7 and 32.8 nm, respectively. Furthermore, their zeta potentials are identified as ‐21.1 and ‐21.5 mV, respectively. Moreover, the polymer core used (PFCN), the amphiphile matrix (CM‐DSPE and PSMA), and the antibody clone (anti‐CYFRA21‐1) are all identical. Nonetheless, the computed solvation energies show that both linkers favor water over n‐octanol. Thus, they are both aqueously stable hydrophilic linkers. Further, when the analytical performance is assessed on the same biotin‐SA test line, P‐3+ yields a background T/C of 0.22. On the contrary, P‐6 gives only 0.07 under identical conditions. Additionally, at 10 ng/mL of CYFRA21‐1 antigen, the two give T/C values of 0.41 and 0.93. The positive‐to‐negative ratios are therefore 1.9 and 13.3. Hence, when only the attachment site differs between the two probes, the observed net 7.1‐fold difference might be attributed solely to orientation.

### 2.4 Test Line Engineering and Synergistic Enhancements

A complete evaluation of antibody orientation, therefore, requires simultaneous consideration of both recognition interfaces in the assay. Nonetheless, the probe‐side measurements described above characterize Fc anchoring, Fab accessibility, and antigen capture in suspension. Meanwhile, they do not capture the second recognition event at the membrane, where a nanoscale probe must interact with an immobilized antibody while retaining access to its surface‐exposed epitope. Moreover, the performance of this second interaction depends on how the capture antibody is presented at the membrane and on the accessibility of the epitope on an already antibody‐decorated particle. Probe‐side metrics are, therefore, necessary but not sufficient to define the optimal assay configuration. Consequently, probe orientation and capture‐antibody orientation cannot be optimized independently, and a full combinatorial screen of all probe configurations against the different test‐line chemistries is required. This approach allows the contribution of each interface to be distinguished and determines whether an orientation that performs well in suspension also remains optimal after membrane capture. The primary metric for resolving these combinations is the positive‐to‐negative (P/N) ratio at 10 ng/mL antigen, which is preferred over the raw T/C ratio because it simultaneously reflects both specific signal and nonspecific background. Hence, a probe with a higher T/C but a lower P/N was rejected.

Test line modification is evaluated independently using all six probes against four nitrocellulose configurations: unmodified (control), protein A‐modified, protein G‐modified, and biotin‐SA‐modified (Figure 3A–D). A comprehensive investigation of optimal probe loading, excitation, and detection parameters, along with the protocol for T/C ratio calculations, has been provided in the Figures S6–S8. Fluorescence imaging of CYFRA21‐1‐positive strips reveals that the unmodified test line generates substantial nonspecific adsorption, manifesting as elevated background at the test line even in the absence of antigen (Figure 3E). Nevertheless, protein A and protein G test lines offer only marginal improvement, with lower positive‐to‐negative (P/N) ratios across most probe configurations (Figure 3F,G). Two factors account for this underperformance. First, optimal antibody binding to protein A and protein G occurs near pH 4.7 [42], well below physiological conditions and incompatible with clinical serum. Second, the capture efficiency of both proteins depends on their own orientation at the nitrocellulose surface, introducing an additional layer of variability.

**FIGURE 3:**
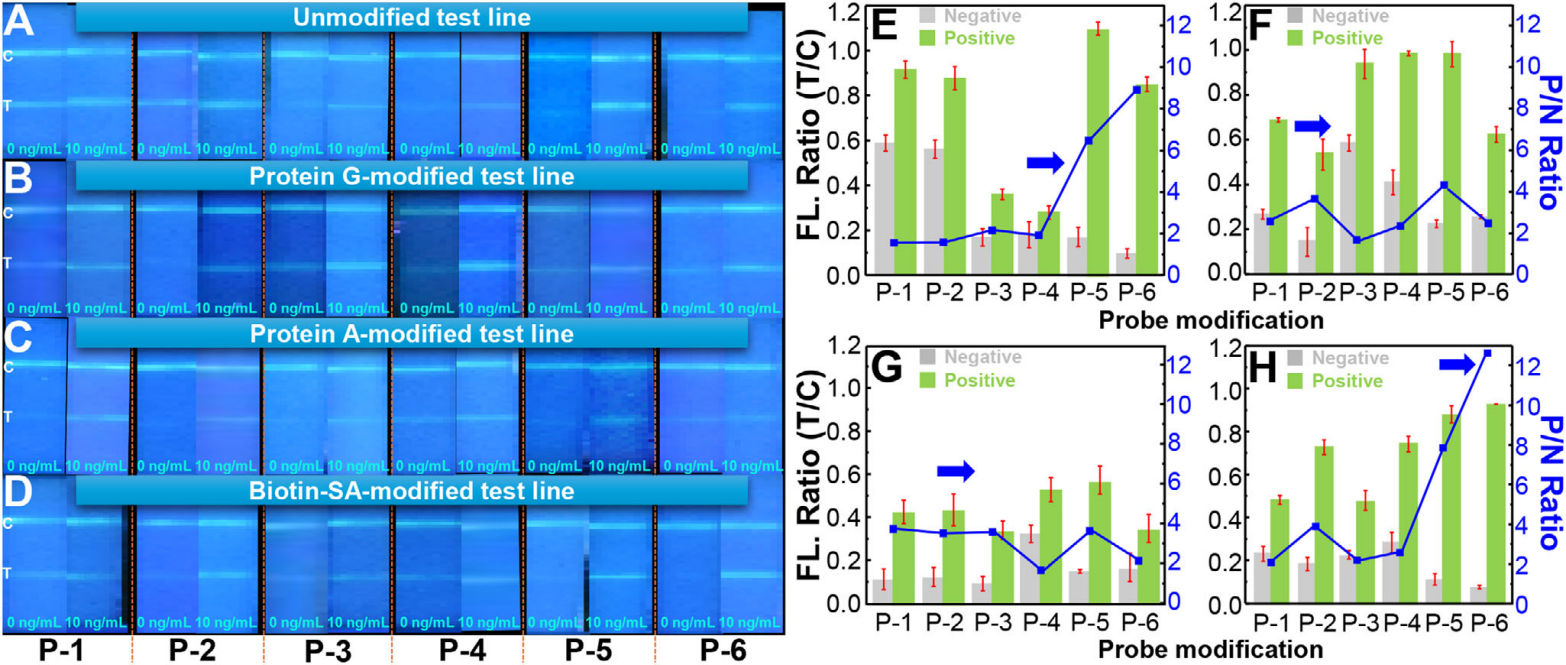
Fluorescence visualization and quantitative analysis of CYFRA21‐1 detection under different test line modification strategies using PFCN‐based P1‐P6 probes. Fluorescence images of (A) unmodified, (B) protein G‐modified, (C) protein A‐modified test line, and (D) biotin‐SA‐modified test lines under UV excitation after reaction with CYFRA21‐1 at 0 and 10 ng/mL. Their corresponding fluorescence ratios of T/C and P/N under different test line configurations: (E) unmodified, (F) protein G modified, (G) protein A‐modified, and (H) biotin‐SA‐modified test lines. Data are mean ± standard deviation of *n* = 3 independent immunoprobe preparations. Each preparation was assayed on a separate test strip. Analysis was performed in ImageJ and OriginPro 9.1. Strip panels are cropped to the nitrocellulose region. The uncropped images are given in Figure S13.

The probe rankings invert across these four test lines (Figure 3E–H). On the unmodified line, P‐6 gives the highest P/N ratio, at about 8.9. On the protein G line, P‐5 gives the highest ratio, at about 4.2, whereas P‐6 reaches only about 2.4. On the protein A line, P‐1 gives the highest ratio, at about 3.7, and P‐6 falls to fifth place at about 2.2. Only on the biotin‐SA line does P‐6 dominate, at about 12.6, against about 8.0 for the next‐best probe. A screen run on a protein A or protein G membrane would therefore be misleading to select a different probe, and the resulting pair would perform about 3‐fold worse than the optimal pair. Hence, the optimal probe is not defined independently of the capture chemistry.

The biotin‐SA‐modified test line produces markedly superior performance across all probe configurations (Figure 3H). This may be inferred due to the biotin‐SA interaction being pH insensitive under physiological conditions, operating with ultrahigh affinity (*K*_d_ near 10^−14^ M) [43], and does not depend on protein surface orientation for function. A PEG_4_ spacer between the biotin handle and the antibody introduces additional linker flexibility, reducing steric crowding at the capture interface. Critically, the enhancement from biotin‐SA modification is most pronounced when combined with the P‐6 immunoprobe (approximately 1.5‐fold higher P/N ratio than the P‐6/unmodified combination), directly demonstrating a synergistic effect between probe‐side and test‐line orientation control. This synergy and inversion of probe rankings has not been previously identified or quantified and constitutes a central finding of this work.

### 2.5 Selectivity Comparison of P‐6/Biotin‐SA‐Modification With P‐3/Unmodified Test Line

Based on the above results, we selected the P‐6 and biotin‐SA pair configuration due to their better control of antibody orientation for subsequent selectivity, sensitivity, multiplexing, and clinical validation studies. We first evaluated its specificity in the LFA platform using various tumor biomarkers with the randomly oriented P‐3 immunoprobe on unmodified test lines serving as the control. As shown in Figure 4A–D, CYFRA21‐1‐functionalized PFCN/P‐3 on unmodified test lines (Figure 4A) and CEA functionalized PFTC6FQ/P‐3 on unmodified test lines (Figure 4B) exhibited apparently poorer detection selectivity compared with their corresponding P‐6 immunoprobes immobilized on biotin‐SA‐modified test lines (Figure 4C,D). The P‐6 probes displayed excellent specificity for CYFRA21‐1 (PFCN) and CEA (PFTC6FQ), with negligible non‐specific adsorption of interfering antigens. Quantitative fluorescence ratios of T/C are summarized in Figure 4E–H. Notably, for PFCN/P‐6, the test line fluorescence for CYFRA21‐1 was more than 4.5‐fold higher than that for other antigens (Figure 4G). Similarly, for PFTC6FQ/P‐6, the fluorescence intensity for CEA exceeded that of other analytes by more than 5‐fold (Figure 4H). These results demonstrate that oriented P‐6 immunoprobes on biotin‐SA‐modified test lines afford substantially enhanced specificity and sensitivity relative to the P‐3/unmodified test line control. Additionally, fluorescence signals for both configurations attained equilibrium within 15 min (Figure 4I), with the P‐6/biotin‐SA system exhibiting superior sensitivity for point‐of‐care applications.

**FIGURE 4:**
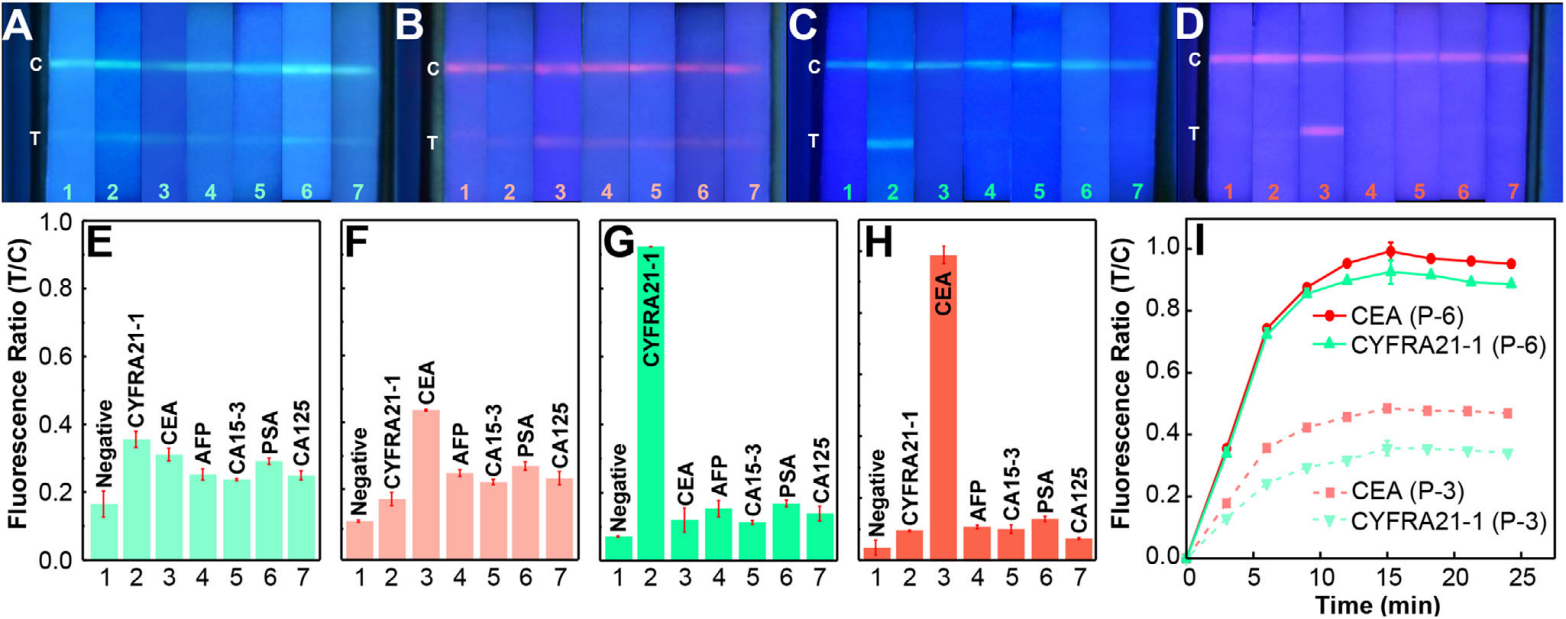
Selectivity evaluation of oriented and randomly oriented immunoprobes based on PFCN and PFTC6FQ Pdots. Oriented probes (P‐6) were immobilized on biotin‐SA modified test lines, while randomly oriented probes (P‐3) were immobilized on unmodified test lines. Test strip images under 365 nm excitation for (A) PFCN/P‐3, (B) PFTC6FQ/P‐3, (C) PFCN/P‐6, and (D) PFTC6FQ/P‐6 after exposure to different antigens (CYFRA21‐1, PSA, and CEA: 10 ng/mL; AFP: 50 ng/mL, CA15‐3 and CA125: 100 U/mL). (E–H) Corresponding T/C fluorescence intensity ratios quantified from (A–D), respectively. (I) Time‐dependent T/C ratios for CYFRA21‐1 (PFCN‐based probes) and CEA (PFTC6FQ‐based probes) using P‐6 and P‐3 conjugation strategies. Data are mean ± standard deviation of *n* = 3 independent immunoprobe preparations. Each preparation was assayed on a separate test strip. Analysis was performed in ImageJ and OriginPro 9.1. Strip panels are cropped to the nitrocellulose region. The uncropped images are given in Figure S14.

### 2.6 Sensitivity Assessment of the P‐6/Biotin‐SA Configuration

Next, we systematically compared the detection sensitivities of randomly oriented P‐3 immunoprobes on unmodified test lines and of oriented P‐6 immunoprobes on biotin‐SA‐modified test lines, with the former serving as the control and the latter as the best pair. Specifically, PFCN/P‐3 Pdots functionalized with CYFRA21‐1 antibodies (Figure 5A) and PFTC6FQ/P‐3 Pdots functionalized with CEA antibodies (Figure 5B) were first evaluated as control configurations on unmodified test lines at increasing analyte concentrations. Pronounced false‐positive responses were observed even in the absence of CYFRA21‐1 or CEA, accompanied by the relatively poor limit of detections (LoDs) of 0.6760 ng/mL for CYFRA21‐1 and 1.129 ng/mL for CEA. When the orientation of the immunoprobe alone was altered by using P‐6 immunoprobes on unmodified test lines (Figures S9 and S10), the LoDs were significantly improved to 0.0969 ng/mL for CYFRA21‐1 and 0.2251 ng/mL for CEA, corresponding to 5‐ to 7‐fold improvements in sensitivity relative to the P‐3 controls. Further integration of test line modification using biotin‐SA chemistry in conjunction with the P‐6 immunoprobes (Figure 5C,D) effectively suppressed background signals to achieve extraordinary LoDs of 0.0352 ng/mL for CYFRA21‐1 and 0.0808 ng/mL for CEA, representing an additional ∼3‐fold improvement compared to P‐6 probes on unmodified test lines. Overall, the combined probe‐ and test line‐manipulation strategies afford cumulative sensitivity enhancements of approximately 19‐fold for CYFRA21‐1 and 14‐fold for CEA relative to the P‐3/unmodified test line configuration. The corresponding calibration plots (Figure 5E‐H) demonstrate comparable dynamic ranges of 0–10 ng/mL for CYFRA21‐1 and 0–15 ng/mL for CEA across all configurations. More precisely, the probe contribution is 6.98‐fold for CYFRA21‐1 and 5.02‐fold for CEA. The test line contributes a further 2.75‐ and 2.79‐fold. Thus, the combined gains are 19.2‐ and 14.0‐fold. Because the two recognition events occur in sequence, these gains multiply to give overall improvements of 19.2‐ and 14.0‐fold. No single‐interface strategy reaches the same limit of detection. This, however, holds well that control of antibody orientation is indeed required at both levels.

**FIGURE 5:**
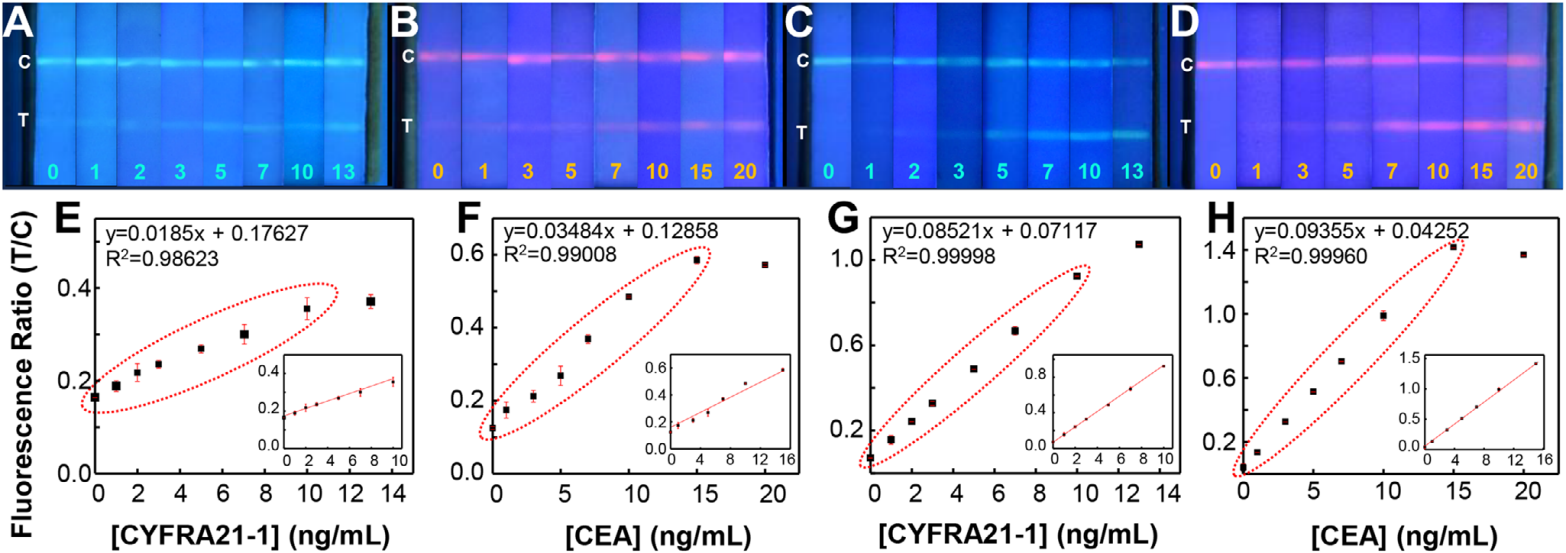
Quantitative fluorescence analysis of CYFRA21‐1 and CEA using different probe orientations and test line modifications. Fluorescence images of test strips under 365 nm light excitation using (A) PFCN/P‐3 and (B) PFTC6FQ/P‐3 probes on unmodified test lines after exposure to CYFRA21‐1 (0‐13 ng/mL) and CEA (0‐20 ng/mL), respectively. Corresponding fluorescence images using (C) PFCN/P‐6 and (D) PFTC6FQ/P‐6 probes on biotin‐SA‐modified test lines. (E–H) Calibration plots and dynamic ranges derived from fluorescence signals for CYFRA21‐1 and CEA, corresponding to panels (A–D), respectively. Data are mean ± standard deviation of *n* = 3 independent immunoprobe preparations. Each preparation was assayed on a separate test strip. Analysis was performed in ImageJ and OriginPro 9.1. Strip panels are cropped to the nitrocellulose region. The uncropped images are given in Figure S15.

### 2.7 Universality of the LFA Platform

To further demonstrate the universality of this antibody‐orientation strategy, the P‐6 modification was extended to construct immunoprobes targeting a diverse panel of clinically relevant tumor biomarkers, which were then benchmarked against the P‐3 control. As schematically illustrated in Figure 6A, the modular immunoprobe design allows the recognition element (e.g., detection antibody here) to be interchanged while preserving the same dual‐site orientation chemistry. It should be noted that biomarker specificity is determined solely by the antigen‐antibody pair selected for each target; this was the only variable adjusted, while the overall orientation framework remained constant. Specifically, six representative tumor biomarkers, including CYFRA21‐1, CEA, AFP, CA15‐3, CA125, and PSA, were selected to cover a broad range of biomolecular sizes and clinical use cases. For each target, the corresponding specific antibody was incorporated into the same P‐6 framework. In parallel, randomly oriented P‐3 immunoprobes carrying identical antibodies were prepared as controls. The randomly oriented P‐3 immunoprobes were evaluated on unmodified test lines, while the orientation‐controlled P‐6 immunoprobes were assessed on biotin‐SA modified test lines under identical assay conditions. As shown in Figure 6B–G, fluorescence images of the test strips revealed that all P‐6/biotin‐SA configurations (right panels) generated much more obvious P/N contrasts on the test lines as compared to the corresponding P‐3/unmodified counterparts (left panels). Besides, occasionally, serious non‐specific adsorption was observed for P‐3/unmodified pairs in the absence of targets. Quantitative analysis of the corresponding T/C and P/N ratios further demonstrates that the P‐6/biotin‐SA immunoprobes could achieve up to a 23.8‐fold enhancement in detection signals relative to the P‐3/unmodified counterparts (Figure 6H). Hence, these findings redefine dual‐site antibody orientation as a key design parameter in LFAs and establish P‐6/biotin‐SA configuration as a generalizable modification for next‐generation rapid‐testing platforms.

**FIGURE 6:**
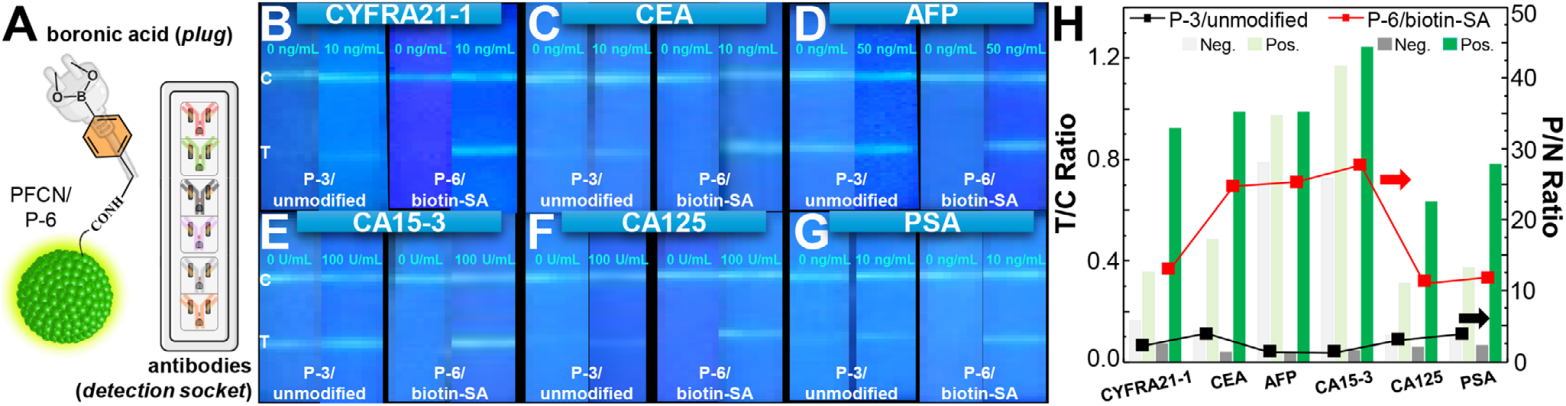
Fluorescence visualization and analytical performance of the universal lateral flow assay platform. (A) Schematic illustrating the universality of the immunoprobe design, (B–G) fluorescence images of test strips acquired under 365 nm excitation for six tumor biomarkers using randomly oriented P‐3 immunoprobes on unmodified test lines and orientation‐controlled P‐6 immunoprobes on biotin‐SA‐modified test lines: (B) CYFRA21‐1 (0 and 10 ng/mL), (C) CEA (0 and 10 ng/mL), (D) AFP (0 and 50 ng/mL), (E) CA15‐3 (0 and 100 U/mL), (F) CA125 (0 and 100 U/mL), and (G) PSA (0 and 10 ng/mL). (H) Corresponding fluorescence T/C and P/N ratios toward various antigens. Data are mean ± standard deviation of *n* = 3 independent immunoprobe preparations. Each preparation was assayed on a separate test strip. Analysis was performed in ImageJ and OriginPro 9.1. Strip panels are cropped to the nitrocellulose region. The uncropped images are given in Figure S16.

### 2.8 Multiplexed Diagnosis of CYFRA21‐1/CEA With P‐6/Biotin‐SA Configuration

We further implemented a spectrally resolved multiplex detection strategy with a P‐6/biotin‐SA configuration to enable simultaneous measurement of CYFRA21‐1 and CEA, thereby improving diagnostic reliability, shortening assay duration, and reducing sample consumption. In multiplexed detection, PFTC6FQ Pdots were designed to detect the target CEA, and PFCN Pdots were used to detect the target CYFRA21‐1. The test strip was patterned with two lines, as shown in Figure 7A, each containing a standard control line and a biotin‐SA‐modified test line co‐immobilized with both CYFRA21‐1 and CEA antibodies. We expect that the CYFRA21‐1 component enables rapid screening for NSCLC while the CEA antibody provides complementary information on other malignancies. In principle, a green, fluorescent signal at the CYFRA21‐1 line indicates caution for NSCLC or progression, whereas a red fluorescent signal from CEA suggests the possibility of other cancers when CYFRA21‐1 is negative. Accordingly, four scenarios are anticipated: (i) no fluorescence for negative samples (Figure 7B), (ii) overlapping red and green signals, appearing yellow, for samples positive for both biomarkers (Figure 7C), (iii) green fluorescence for CYFRA21‐1 positive samples (Figure 7D), and (iv) red fluorescence for CEA positive samples (Figure 7E). The results suggest that the probes and test line are highly selective and exhibit minimal cross‐reactivity, making them suitable for multiplexed detection in complex biological matrices.

**FIGURE 7:**
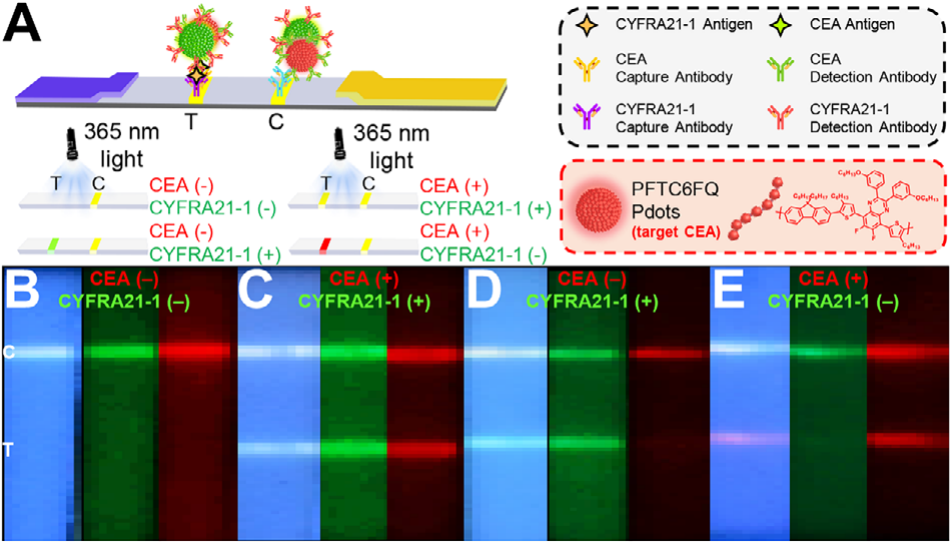
CYFRA21‐1 and CEA‐based multiplexed detection in serum samples. (A) Schematic of the LFA multiplex detection platform used in the study. (B–E) Photographs of test strips taken under 365 nm light without any optical filter (left panel), with a 525 ± 25 nm band‐pass filter (middle panel), and with a 680‐IR nm band‐pass filter (right panel), where the samples contain CEA and CYFRA21‐1 of (B) 0 and 0 ng/mL, (C) 10 and 10 ng/mL, (D) 0 and 10 ng/mL, and (E) 10 and 0 ng/mL, respectively. Strip panels are cropped to the nitrocellulose region. The uncropped images are given in Figure S17.

### 2.9 Clinical Validation of CYFRA21‐1/CEA in Lung Cancer Patients

The translational readiness of the platform is assessed using serum samples from seven healthy volunteers and 21 patients representing diverse histological subtypes (Figure 8): “squamous cell carcinoma” (2 patients), ″adenocarcinoma’ (15 patients), “small cell lung carcinoma” (3 patients), and “invasive mucinous adenocarcinoma” (1 patient). Patient values span 0.47 to 14.13 ng/mL for CYFRA21‐1. They span 1.10 to 16.30 ng/mL for CEA. Healthy values span 0.62 to 2.01 ng/mL and 0.71 to 1.91 ng/mL. The P‐6/biotin‐SA LFA platform yields quantitative fluorescence intensities that strongly correlate with clinically determined concentrations measured by electrochemiluminescence immunoassay (ECLIA) at the hospital (Table 2). Healthy volunteer measurements show mean absolute deviations of less than 0.1 ng/mL from ECLIA values. Patient measurements show close agreement across all histological subtypes and concentration ranges, with the LFA‐derived values falling within approximately 8% (Figure 9). Moreover, the mean absolute deviation for patients stays below 0.07 ng/mL for both markers.

**FIGURE 8:**
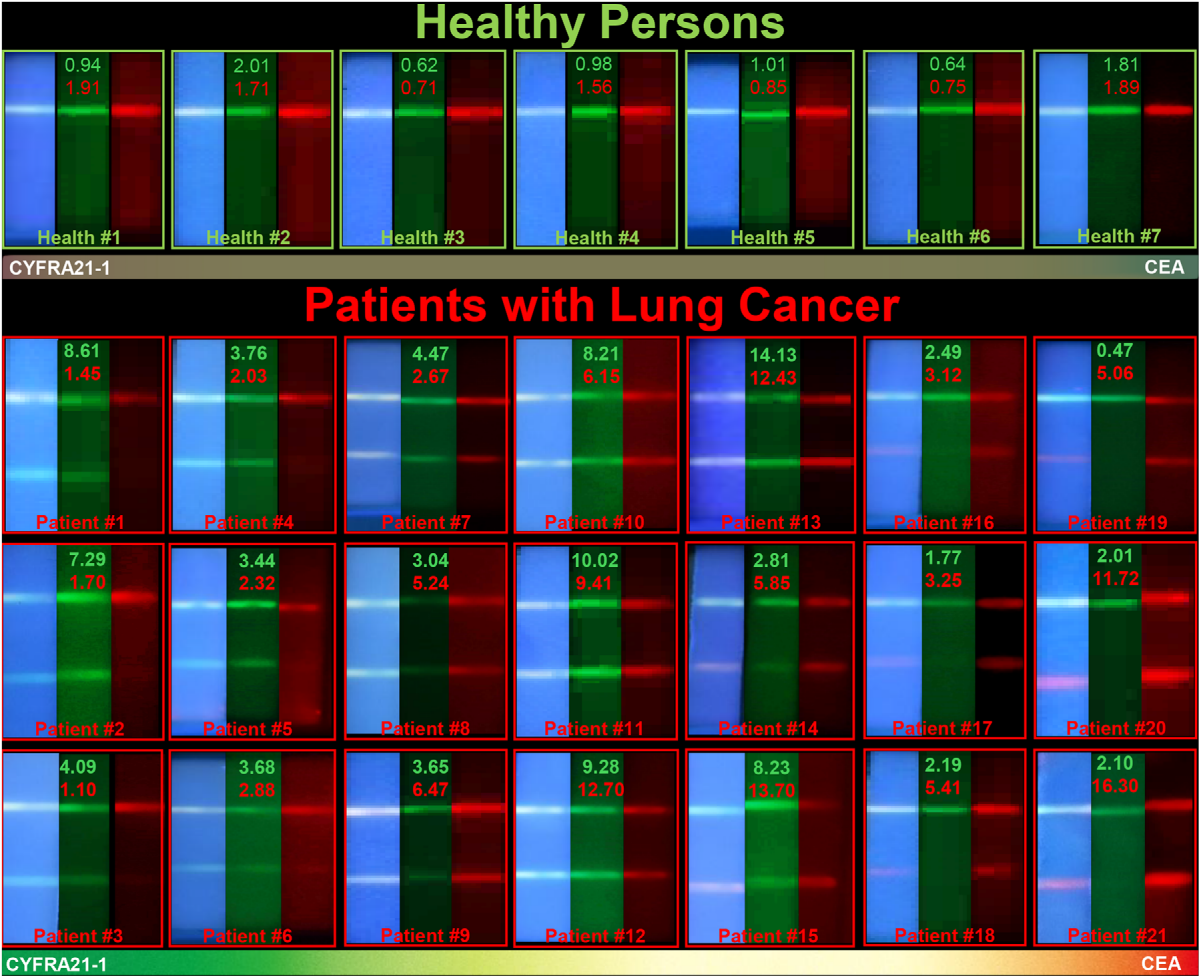
Clinical validation of the platform containing seven healthy volunteers and 21 patients with different types of lung cancer by the P‐6/biotin‐SA configuration. Test strips were photographed under 365 nm light without an optical filter (left panel), with a 525±25 nm band‐pass filter (middle panel), and with a 680‐IR nm band‐pass filter (right panel). The test results from an “electrochemiluminescence immunoassay (ECLIA)” are displayed by the numbers on the test strips. The green numbers represent CYFRA21‐1 levels (ng/mL), and the red numbers represent CEA levels (ng/mL). The uncropped images are given in Figure S18.

**TABLE 2:** Values of CYFRA21‐1 and CEA in the 28 clinical samples measured by ECLIA and by this Pdot‐based LFA platform.

Sample	CYFRA21‐1^a^ (ng/mL)	CEA^a^ (ng/mL)	Histology	CYFRA21‐1^b^ (ng/mL)	CEA^b^ (ng/mL)
Health #1	0.94	1.91	Healthy	0.83	1.74
Health #2	2.01	1.71	Healthy	1.89	1.63
Health #3	0.62	0.71	Healthy	0.67	0.75
Health #4	0.98	1.56	Healthy	0.91	1.43
Health #5	1.01	0.85	Healthy	0.98	0.90
Health #6	0.64	0.75	Healthy	0.62	0.87
Health #7	1.81	1.89	Healthy	1.82	1.86
Patient #1	8.61	1.45	Squamous cell carcinoma	8.58	1.51
Patient #2	7.29	1.70	Adenocarcinoma	7.31	1.66
Patient #3	4.09	1.10	Adenocarcinoma	4.07	1.03
Patient #4	3.76	2.03	Squamous cell carcinoma	4.04	2.19
Patient #5	3.44	2.32	Adenocarcinoma	3.47	2.35
Patient #6	3.68	2.88	Adenocarcinoma	3.71	2.92
Patient #7	4.47	2.67	Adenocarcinoma	4.48	2.62
Patient #8	3.04	5.24	Adenocarcinoma	3.11	5.22
Patient #9	3.65	6.47	Adenocarcinoma	3.86	6.71
Patient #10	8.21	6.15	Adenocarcinoma	8.22	6.16
Patient #11	10.02	9.41	Adenocarcinoma	9.94	9.33
Patient #12	9.28	12.70	Small cell lung carcinoma	9.33	12.74
Patient #13	14.13	12.43	Adenocarcinoma	14.08	12.56
Patient #14	2.81	5.85	Adenocarcinoma	2.77	5.84
Patient #15	8.23	13.70	Adenocarcinoma	8.26	13.66
Patient #16	2.49	3.12	Small cell lung carcinoma	2.55	3.12
Patient #17	1.77	3.25	Adenocarcinoma	1.79	3.22
Patient #18	2.19	5.41	Small cell lung carcinoma	2.07	5.35
Patient #19	0.47	5.06	Adenocarcinoma	0.44	5.10
Patient #20	2.01	11.72	Invasive mucinous adenocarcinoma	1.97	11.56
Patient #21	2.10	16.30	Adenocarcinoma	2.10	16.27

^a^ Clinical samples determined by ECLIA,

^b^ Clinical samples determined by fluorescence LFA platform.

**FIGURE 9:**
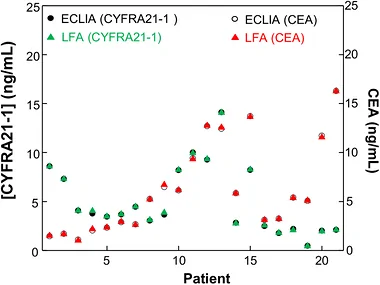
Quantitative determination of CYFRA21‐1 and CEA values in clinical serum samples collected from 21 patients (#1‐21) with different types of lung cancer. Data from ECLIA (black circles) and P‐6/biotin‐SA modification‐based LFA (red and green triangles) were analyzed. Each sample was measured once by each method, for a total of 28 subjects. Analysis was performed in OriginPro 9.1.

Clinical serum is a strongly interfering matrix, so matrix‐matched calibrations were also made. CYFRA21‐1 was spiked into pooled human serum at four dilution levels (Figure S19). In undiluted serum, the T/C ratio stays at zero at every concentration tested. We attribute this complete blocking to three effects acting together: i) serum protein passively adsorbs to the membrane and forms a corona on the probe, ii) the higher viscosity of serum also slows capillary transport to the test line, and iii) serum absorbance and autofluorescence attenuate both excitation and emission. At a 3‐fold dilution, the signal returns, but the fit remains poor with a slope of 0.0877 and an *R* ^2^ of 0.9387. However, at 5‐ and 10‐fold dilutions, the fits are excellent. Both fits yield an *R* ^2^ of 0.9999, and their slopes are 0.0852 and 0.0863, respectively. Hence, the clinical samples in this work were diluted 5‐ to 20‐fold into the running buffer. Most importantly, dilution brings each sample into the assay's linear range since the clinical marker levels span more than two orders of magnitude. Dilution also guards against signal loss at very high antigen levels. Notably, the buffer limits of detection propagate through this dilution range. They give serum detection limits of 0.176 to 0.704 ng/mL for CYFRA21‐1, and 0.404 to 1.616 ng/mL for CEA. Since the clinical decision thresholds are 3.3 and 5.0 ng/mL for CYFRA21‐1 and CEA, respectively, the platform maintains margins of at least 4.7‐ and 3.1‐fold, respectively. Therefore, the significance of the dual‐site strategy is clearly highlighted in this comparison. The conventional P‐3 probe on an unmodified line has limits of 0.6760 and 1.129 ng/mL, which become 3.38 to 13.52 ng/mL and 5.65 to 22.58 ng/mL after the same dilution. Unfortunately, both lie above the clinical thresholds at every dilution used here. Hence, that configuration is therefore blind at the clinical decision point. Moreover, probe side orientation alone recovers only a marginal window. Its CEA limit is just 1.1‐fold below the threshold at a 20‐fold dilution. On the contrary, only the dual‐site configuration maintains a usable margin across the entire range. Dual site orientation is therefore not simply an incremental gain in sensitivity. It is what enables measurement in diluted clinical serum. Since these results were achieved without any laboratory instrumentation, specialist personnel, or controlled ambient conditions, this highlights the practical suitability of this platform for real‐world point‐of‐care deployment.

## 3 Conclusion

We have demonstrated that the rational engineering of antibody orientation at two interfaces of a lateral flow immunoassay (the nanoprobe and the test line) produces sensitivity improvements that exceed those achievable with any single‐interface approach. The dual site framework delivers 19‐ and 14‐fold LOD improvements for CYFRA21‐1 and CEA, respectively, relative to the conventional EDC/NHS benchmark. The probe contribution is 6.98‐fold for CYFRA21‐1 and 5.02‐fold for CEA. The test line contributes a further 2.75‐ and 2.79‐fold. Because the two recognition events occur in sequence, these contributions multiply to give the overall gains (Table S1). Moreover, the probe rankings also invert across capture chemistries. The best probe, therefore, cannot be chosen independently of the test line. Critically, the platform requires no modification of the antibody, the antigen, or the nitrocellulose substrate; only the surface chemistry of the probe and the test line linker need to be modified. Four independent and convergent characterization methods (Fc quantification, BCA antigen binding, SDS‐PAGE under reducing conditions, and FRET lifetime analysis) establish P‐6 as the optimal probe architecture. The mechanistic framework developed here explains why para‐substituted boronic acid outperforms meta‐substituted boronic acid, protein A/G affinity binding, and biotin‐SA coupling with non‐regioselective biotinylation: the para isomer with its methylene spacer maximizes geometric access to the Fc glycan while minimizing steric strain on the boronate ester bond. The framework is universal and functions across six clinically relevant tumor biomarkers spanning a wide range of molecular sizes and clinical contexts solely through antibody substitution, without changes to surface chemistry. The developed LFA platform also supports spectrally resolved multiplex detection of two co‐occurring NSCLC biomarkers on a single strip, and it has been validated in 28 clinical serum samples against ECLIA, with patient values agreeing to within about 8%. The reported quantity is nevertheless independent of the detector. The T/C ratio is internally referenced within a single image frame. Both lines are imaged through the same optics under the same illumination. Therefore, detectors gain and exposure cancel in the ratio. We find the T/C ratio to be unchanged across the two smartphones and the camera (Figure S7), where only the signal‐to‐noise ratio differs between the three detectors. Thus, moving the readout to a smartphone requires no change to the assay.

In addition, this detector‐independent architecture provides a practical foundation for the next generation of AI‐integrated point‐of‐care diagnostics. Particularly, smartphone imaging can be coupled with machine‐learning algorithms for automated signal extraction, quantitative interpretation, and potentially decentralized clinical decision support [44, 45]. Even though an algorithm can recover a signal from a noisy image, it cannot recover a binding event that never happened. The future of rapid diagnostics, therefore, lies not in AI alone, but in the integration of reliable assay chemistry, detector‐independent quantitative readout, and AI‐assisted interpretation within a single point‐of‐care platform.

Beyond antibody‐based LFAs, the dual‐site orientation principle may also be extended to nucleic‐acid‐ and aptamer‐based recognition systems. In these assays, the recognition element is likewise presented at two sequential interfaces: the reporter probe must retain sufficient structural accessibility for target recognition, while the capture element on the test line must remain accessible to efficiently immobilize the probe‐target complex. Recent studies have demonstrated that DNA nanostructures can improve the ordered presentation and stability of nucleic acid recognition elements on lateral‐flow membranes [46], while aptamer‐based LFAs have employed independently functionalized capture and reporter aptamers to achieve sandwich recognition without relying on antibody‐protein interactions [47]. Multisite bridging and DNA tetrahedron architectures have further demonstrated that increasing the number and accessibility of nucleic acid binding sites can enhance probe capture and signal generation at the test line [48, 49]. These findings suggest that, although the specific chemistry must be adapted to the structural properties of each recognition element, the underlying design principle established here (simultaneous control of recognition‐element presentation at both the probe and capture interfaces) could provide a general framework for improving sensitivity and reliability across antibody‐, aptamer‐, and nucleic‐acid‐based LFAs. Taken together, these results collectively establish dual‐site antibody orientation control as a fundamental design principle, rather than an empirical optimization step, for the next generation of point‐of‐care diagnostic platforms.

## 4 Experimental Section

### 4.1 Preparation of Pdots

“PFTC6FQ,” “PFCN,” “carboxymethyl PEG‐DSPE” (CM‐DSPE, *M*_w_ = 2000), and “cumene‐terminated poly(styrene‐co‐maleic anhydride)” (PSMA, Mn≈ 1900; 75% styrene content) were individually prepared as stock solutions in THF at a concentration of 1 mg/mL. Subsequently, a homogeneous organic phase was obtained by sequentially combining 100 µL of either PFCN or PFTC6FQ in a 20 mL glass vial with 15 µL of CM‐DSPE and 7 µL of PSMA containing 2.5 mL of THF. Meanwhile, 5 mL of deionized water was subjected to continuous ultrasonication in a separate glass container, and the organic mixture was rapidly introduced into the aqueous phase. The resulting dispersion was heated at 70°C under nitrogen for approximately 20 min to ensure complete evaporation of THF, producing an aqueous Pdot suspension with a final volume of approximately 4 mL. After cooling to ambient temperature, the suspension was passed through a syringe filter containing a “0.22 µm cellulose acetate membrane” to eliminate large‐sized aggregates.

### 4.2 Surface Modification of Pdots

Six variants of surface‐engineered Pdots (P‐1 to P‐6) were fabricated through different conjugation approaches. For the preparation of P‐1, 2 mL of the Pdot suspension was combined with HEPES buffer (40 µL, 1 M, pH = 7.4), EDC solution (40 µL, 5 mg/mL, freshly prepared), NHS solution (10 µL, 5 mg/mL, freshly prepared), PEG solution (40 µL, 5% w/w, *M*_n_ = 3350), and protein A solution (200 µL, 1 mg/mL). To collect the functionalized Pdots, the suspension was gently mixed at room temperature for 3.5 h and centrifuged for 10 min at 2500 rpm. An identical protocol was applied for P‐2, with protein A replaced by protein G (200 µL, 1 mg/mL). For P‐4 to P‐6, surface functionalization was achieved by substituting the protein component with 50 µL of 3‐aminophenylboronic acid (1 mg/mL), 200 µL of streptavidin (1 mg/mL), or 50 µL of 4 (aminomethyl)phenylboronic acid (1 mg/mL), respectively, under the same coupling conditions. All functionalized Pdots were stored at 4°C for short‐term use or purified further by Sephacryl S‐300 HR via size‐exclusion chromatography using 5% (w/w) PEG with 20 mM HEPES (pH = 7.4) as the eluent for long‐term storage.

### 4.3 Antibody Conjugation of the Functionalized Pdots

For CYFRA21‐1 antibody immobilization, 2 µL of anti‐CYFRA21‐1 (1 mg/mL, product code 10–2461, Biosynth) was combined with 200 µL of the corresponding surface‐modified PFCN Pdots (P‐1, P‐2, P‐4, or P‐6) and incubated for 1.5 h in a thermostated water bath at 37°C. For P‐3, Pdots were initially activated via EDC/NHS coupling, as described above, followed by direct conjugation with 2 µL of CYFRA21‐1 antibody at ambient temperature for 3.5 h; the resulting conjugates were isolated by centrifugation for 10 min at 2500 rpm. For P‐5, antibodies were first derivatized with biotin‐PEG4‐amine through NHS‐ester chemistry and subsequently immobilized onto streptavidin‐functionalized Pdots. The antibody‐conjugated Pdots were either employed immediately or stored at 4°C prior to use. For CEA antibody conjugation, 2 µL of CEA antibody (1 mg/mL, product code 10–7882, Fitzgerald, USA) was mixed with 200 µL of functionalized PFTC6FQ Pdots (P‐6) and incubated under identical conditions. For P‐3, a similar procedure was carried out as CYFRA21‐1 conjugation.

### 4.4 Materials

All chemicals were of analytical grade and used as received unless otherwise noted. Highly pure water (18.2 MΩ. cm) was used throughout our experiments. Semiconducting polymers PFCN [50], and PFTC6FQ [51] were synthesized according to the reported literature. CYFRA21‐1 antibodies (10‐2461 and 10–2462, Biosynth, USA), CEA antibodies (10‐7882 and 10–7883, Fitzgerald, USA), AFP antibodies (10‐1390 and 10–1391, Biosynth, USA), CA15‐3 antibodies (136549 and 136551, United States Biological Corporation, USA), CA125 antibodies (10‐C02F, Biosynth, USA; 10‐C02E, Fitzgerald, USA), and PSA antibodies (10‐P20D and 10‐P20E, Biosynth, USA), were used as received. CYFRA21‐1 antigen (30‐AC69, Fitzgerald, USA), CEA antigen (30‐AC26, Biosynth, USA), AFP antigen (30‐1029, Fitzgerald, USA), CA15‐3 antigen (C0050‐27, United States Biological Corporation, USA), CA125 antigen (30‐AC21, Fitzgerald, USA), and PSA antigen (30C‐CP1017U, Fitzgerald, USA) were diluted up to desired concentrations by PBS buffer upon use and used without further purification. Protein A (PB0789, Bio Basic Inc., Canada) and protein G (P6872, Bio Basic Inc., Canada) were prepared in ultrapure distilled water, where biotin‐PEG4‐amine (QI‐0446, Combi‐Blocks, Inc., USA) and streptavidin (pro‐791‐b, ProSpec, Israel) were prepared as solutions in 20 mM HEPES (pH = 7.4). Furthermore, FITC, 3‐aminophenylboronic acid, and 4‐(aminomethyl)phenylboronic acid were prepared in 10 mM HEPES (pH = 8.5). Nitrocellulose membranes (5 µm, CNPC), sample pads (GFB‐R4), and absorbent pads (AP080) were obtained from mdi Membrane Technologies. These materials were then cut to the desired dimensions to assemble test strips, which were subsequently inserted into 4‐mm plastic cassettes.

### 4.5 Evaluation of Antibody Orientation

#### 4.5.1 Quantification of unanchored Fc regions of Ab

FITC (0.5 mg/mL) was reacted with 4‐(aminomethyl)phenylboronic acid (0.19 mg/mL) at a 1:1 molar ratio at room temperature for 1.5 h to obtain FITC‐labeled boronic acid. Subsequently, 180 µL of each of the six immunoprobes was separately mixed with 20 µL of FITC‐labeled boronic acid and incubated at room temperature for 1–2 h. The mixtures were then centrifuged at 2500 rpm for 10 min using a 100 kDa MWCO membrane filter, and the absorbance of the collected supernatant was measured. The concentration of unbound FITC‐labeled boronic acid (*C*_unbound_) in the supernatant and the total FITC before centrifugation (*C*_total_) was determined from a linear calibration curve (*y* = *mx* + *c*), using *x* = (*y*‐*c*)/*m*. The amount of bound reporter was subsequently calculated by mass balance according to *C*_bound_ = (*C*_total_─*C*_unbound_)/*C*_total_ and expressed as a percentage. This value directly reflects the fraction of unanchored Fc domains accessible for boronic acid binding.

#### 4.5.2 BCA Analysis

To evaluate the antigenic activity of the six immunoprobes, 180 µL of CYFRA21‐1 antigen solution (10 µg/mL) was mixed with 20 µL of each immunoprobe, and the mixtures were incubated at 37°C for 1 h. The reaction mixtures were then centrifuged at 2500 rpm for 10 min. Following centrifugation, the unbound antigen in the supernatant is quantified by bicinchoninic acid (BCA) colorimetry, and the captured antigen is calculated by the difference (*C*_captured_ = *C*_total antigen added—_ *C*_supernatant_ = 10 – *C*_supernatant_).

#### 4.5.3 SDS‐PAGE Analysis

The orientation of the antibody on each immunoprobe was assessed under reducing conditions. Briefly, each immunoprobe (P‐1 to P‐6) and free anti‐CYFRA21‐1 antibody (Ab, as control) were first treated with dithiothreitol (DTT, 50 mM final concentration) at 95°C for 10 min to cleave inter‐chain disulfide bonds, liberating heavy chains (HC, ∼50 kDa) and light chains (LC, ∼25 kDa). The reduced samples were mixed with 4× Laemmli sample buffer and resolved on a 10% gradient SDS‐PAGE gel at 120 V for 90 min. Following electrophoresis, the gel was stained with Coomassie Brilliant Blue R‐250 for 1 h and destained overnight. Band intensities of the HC (∼50 kDa) region were quantified using ImageJ. For probes in which the antibody HC is anchored to the Pdot surface via the Fc glycan (as in P‐6), the HC remains tethered to the nanoparticle and is retained in the sample well during electrophoresis, resulting in a markedly diminished HC band relative to the free‐antibody control. The relative HC band intensity thus serves as an inverse indicator of Fc‐directed orientation efficiency across all six probes.

#### 4.5.4 FRET Experiments

FITC (0.5 mg/mL, 40 µL) was directly conjugated to CYFRA21‐1 antibodies (2 µL) by incubation at 37°C in a water bath for 1 h. Excess FITC was removed by centrifugation at 2500 rpm for 10 min using a 30‐kDa molecular‐weight cutoff (MWCO) membrane filter. To functionalize the FITC‐labeled CYFRA21‐1 antibodies onto P‐4 and P‐6‐modified PPE polymer dots (Pdots), 2 µL of FITC‐labeled antibodies was mixed with 200 µL of the corresponding functionalized Pdots and incubated at 37°C for 1.5 h in a water bath.

For the P‐3 immunoprobes, Pdots were first activated using EDC/NHS chemistry and subsequently reacted with 2 µL of CYFRA21‐1 antibodies at room temperature for 3.5 h. The resulting conjugates were purified by centrifugation at 2500 rpm for 10 min using a 100 kDa MWCO membrane filter. PPE Pdots were both prepared and functionalized identically to the PFTC6FQ and PFCN Pdots.

#### 4.5.5 Calculating the Förster Radius, *R*_0_

FRET is a nonradiative dipole‐dipole interaction in which the Förster radius (*R*_0_) represents the donor–acceptor separation distance at which the energy transfer efficiency is 50%. The Förster distance provides critical insight into the intrinsic efficiency of a given donor‐acceptor pair. In this study, the Förster radius was calculated using the following equation:

(1) $$R_{0}=0.211\times \left[k^{2}n^{-4}{\phi_{D}}^{2}J\right]\left(\mathrm{in}\;Å\right)$$

where *k* ^2^ is the dipole orientation factor, taken as 2/3 for a randomly oriented donor‐acceptor system; *n* is the refractive index of the medium (1.33 for aqueous systems); *ϕ*_D_ = quantum yield of the donor in the absence of the acceptor (0.12 for PPE Pdots), and *J* is the spectral overlap integral describing the extent of overlap between the donor emission spectrum and the acceptor absorption spectrum.

The spectral overlap integral *J* was calculated using the following expression:

(2)

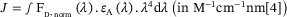

where *F*_D_, _norm_(*λ*) = normalized donor emission intensity, *λ* is the wavelength (nm), and *ε*_A_(*λ*) = molar absorption coefficient of the acceptor at *λ*, which is 7 × 10^4^ M^−1^ cm^−1^ for FITC. Based on these parameters, the calculated spectral overlap integral was 5.52 × 10^17^ M^−1^cm^–^1nm [4]. On substituting the value of J in Equation (1), we get *R*_0_ = 10.53 nm. Thus, it is evident that even with a lower quantum yield, the conjugated polymer's overlap integral is sufficiently strong to maintain a sensing range of over 10 nm.

*Theoretical Investigations*: All theoretical calculations, including geometry optimization of the structure, were performed at the B3LYP/6‐31G+ (d,p)‐SDD level with the Gaussian 16 W software suite.

#### 4.5.6 Characterization of the Immunoprobes

The UV‐visible spectra of the obtained nanohybrids were characterized using a UV–visible spectrophotometer (DB20S, Dynamica Limited). The average hydrodynamic radius was measured by the dynamic light scattering (DLS) instrument (Malvern Zetasizer Nano S), and TEM images of the as‐prepared Pdots were measured by a Hitachi transmission electron microscope (HT7700) at an acceleration voltage of 100 kV. The preparation of the TEM sample involved carefully depositing 8 µL of Pdot aqueous solution (6‐fold diluted) onto a carbon‐coated grid and placing the grid in a dry cabinet for 24 h. The grid was further placed inside an oven at 80°C for an additional 24 h to ensure the water was completely evaporated. The fluorescence spectra were acquired on an FS5 spectrofluorometer (Edinburgh Instruments Ltd.). Fourier transform infrared spectra (FTIR) of the Pdots and immunoprobes were obtained using a PerkinElmer Spectrum 100 FTIR instrument.

#### 4.5.7 Agarose Gel Electrophoresis

Agarose gel electrophoresis was performed to confirm the successful surface functionalization of the immunoprobes. A 1% (w/v) agarose gel was prepared in 60 mL of HEPES buffer (20 mM, pH 7.4). Each sample (15 µL of bare PFCN Pdots or P‐1 to P‐6 immunoprobes, normalized to an equal particle concentration) was mixed with 2 µL of 6× loading buffer (2.5% Ficoll‐400, 10 mM EDTA, 3.3 mM Tris‐HCl, 0.08% SDS, 0.02% Dye 1, and 0.001% Dye 2, pH 8.0) and loaded into the gel wells. Electrophoresis was carried out at 50 V for 60 mins in 350 mL of HEPES running buffer (20 mM, pH 7.4) at room temperature. The gel was then illuminated with a UV flashlight (365 nm) and imaged with a digital camera using a band‐pass filter of 525±25 nm. The electrophoretic mobility shift of each immunoprobe relative to bare PFCN Pdots confirms a change in surface charge and hydrodynamic size arising from the respective surface modification.

#### 4.5.8 Preparation of the Test Strips by Using the Immunoprobes

The sample pad and absorbent pad were cut into squares measuring 6 mm (length) x 3 mm (width). The nitrocellulose membrane (3 mm in width) was fabricated with a test line composed of either CYFRA21‐1 (10‐2462, 0.15 mg/mL), CEA (10‐7883, 0.7 mg/mL), AFP (10‐1391, 0.3 mg/mL), CA15‐3 (136551, 0.3 mg/mL), CA125 (10‐C02E, 0.3 mg/mL), or PSA (10‐P20E, 0.15 mg/mL) antibodies, and a control line composed of IgG secondary antibodies (1 mg/mL) by using an automated lateral flow reagent dispenser. For multiplexed detection, the test line was loaded with both CYFRA21‐1 and CEA capture antibodies together. The test strip was placed under a vacuum for 20 min to dry it. After fabrication of the test and control lines, the test strip was assembled by integrating the absorbent pad with the sample pad onto the nitrocellulose membrane. The test strip was then inserted into a 4‐mm plastic cassette.

#### 4.5.9 Detection of CYFRA21‐1/CEA by P‐6 Immunoprobe

To prepare the running buffer, various substances were combined. Specifically, 10 𝜇L of 5% (w/w) Triton X‐100, 5 𝜇L of 1% (w/w) glycerol, and 5 𝜇L of 5% (w/w) polyethylene glycol 3000 were mixed. Additionally, 1 𝜇L of P‐6 immunoprobe, X 𝜇L of the target solution containing CYFRA21‐1 and/or CEA, 5 𝜇L of serum, and (74‐X) 𝜇L of 20 mM HEPES buffer (pH = 7.4) were added to the mixture. For the calibration samples used to establish the calibration curves, CYFRA21‐1/CEA were spiked into the running buffer. Volumes ranging from 0 to 20 𝜇L of the spiked solution were added to the running buffer, resulting in a final volume of 0.1 mL. For testing human serum samples, patient samples were diluted several times to ensure biomarker levels fell within the assay's linear dynamic range. The fluorescence images of the test strips can be captured using a regular digital camera (e.g., Nikon D5500) or a smartphone under a handheld 365 nm UV flashlight (model SV‐13, 20 W, HaixnFire International, LLC). The camera setting is: 1) F‐stop: f/3.5; 2) Exposure time: 1/15‐1/25 s; 3) ISO speed: ISO‐1000; 4) Exposure bias: ‐1 step; 5) Focal length: 18 mm; 6) Max aperture: 3.6; 7) Metering mode: Multi‐zone; 8) Flash mode: No flash; 9) 35 mm focal length: 27; 10) Color temperature: 4000 K. Subsequently, these images were processed using ImageJ with Java to measure the fluorescence ratios between the control and test lines. The detailed detection of clinical samples by our LFA platform is presented in the Videos S1 and S2.

The probe loading volume was optimized by evaluating fluorescence T/C and S/N ratios at 0.5, 1, 3, 5, and 7 µL (Figure S8). While the T/C ratio was insensitive to probe concentration, the S/N ratio was maximized at 1 µL; higher volumes yielded progressively higher background fluorescence due to excess unbound probe. Accordingly, 1 µL of immunoprobe was adopted for all experiments reported herein. To establish standardized imaging conditions, we systematically evaluated excitation wavelength (254, 365, and 395 nm), lamp power (4, 15, and 20 W), detector type (Android smartphone, iPhone, and NIKON DSLR), and excitation duration (15‐75 s) (Figure S9). The T/C ratio was insensitive to all parameters tested, whereas the S/N ratio was optimized at 365 nm excitation, 20 W power, and a NIKON D5500 digital camera. The T/C ratio was stable across all exposure durations, confirming that 15s illumination is sufficient for robust quantitative readout.

#### 4.5.10 Ethics Statement and Human Subjects

All work with human serum followed the Declaration of Helsinki. The study was approved by the Research Ethics Committee of National Taiwan University Hospital. The approved study case number is 202410032RINC. Written informed consent was obtained from every participant before collection. The committee approved the consent document, version 5.0, dated 8 November 2024. Residual serum from routine clinical blood draws was used throughout. Samples were de‐identified and coded before transferring to the laboratory. No identifying information was available to the analysts at any stage. Healthy volunteer samples were collected under the same approved protocol. The collection took place at the National Taiwan University Hospital, Hsinchu Branch. Samples were collected between February 2025 and December 2025.

#### 4.5.11 Preparation of the P‐3+ Control Probe

Thiol‐terminated PFCN Pdots were prepared by nanoprecipitation as above. Poly(styrene‐co‐thiol) replaced part of the PSMA in the organic phase. The CM‐DSPE amphiphile content was left unchanged. Separately, 2 µL of anti‐CYFRA21‐1 at 1 mg/mL was reacted with SMCC. A 5‐fold molar excess was used for 2 h at room temperature. The activated antibody was added to 200 µL of the thiol‐terminated Pdots. The mixture was reacted for 3.5 h at 200 rpm and room temperature. The conjugate was collected by centrifugation at 2500 rpm for 10 min. All subsequent handling matched that used for the other immunoprobes.

#### 4.5.12 Antibody Loading as a Function of pH

Anti‐CYFRA21‐1 was conjugated to P‐4‐ and P‐6‐functionalized PFCN Pdots. Conjugation buffers were 20 mM HEPES at pH 6.5, 7.0, 7.5, 8.0, and 8.5. Antibody was offered at 1.00 mg/mL in every case. Three independent replicates were prepared at each pH value. Incubation was for 1.5 h at 37 C in a thermostated bath. The conjugates were then collected by centrifugation steps. Unbound antibody in the supernatant was quantified by BCA assay. The calibration curve used the same antibody across 0.1–1.1 mg/mL. The amount immobilized was then obtained by difference. All other conjugations in this work were performed at pH 7.4.

#### 4.5.13 Matrix Matched Calibration in Human Serum

Pooled human serum was spiked with CYFRA21‐1 across the working range. Samples were assayed with the P‐6 and biotin‐SA configuration. They were run neat and at 3‐, 5‐, and 10‐fold dilutions. Dilution was into the standard running buffer described above. A calibration curve was constructed at each dilution level. Each curve was compared with the curve obtained in buffer alone. Clinical serum samples were diluted 5‐ to 20‐fold before application. The factor for each sample kept the reading within the linear range.

#### 4.5.14 Computational Methods

Conformer sampling used CREST v3.0.2 for each boronic acid linker [52]. The level was GFN2‐xTB//GFN‐FF with ALPB implicit water solvation [53]. The command was a crest input.xyz –chrg 0 –alpb water ‐T 8. The conformer energy window was 6.0 kcal/mol. The RMSD threshold was 0.25 Å, and the coordination‐number cutoff was 0.30. Final CREGEN clustering used an RMSD threshold of 0.125 Å, a rotational constant threshold of 0.01, and an energy threshold of 0.05 kcal/mol. Populations are those reported by CREST at 298.15 K. All further structures were optimized by density functional theory. The level was B3LYP/6‐31+G(d,p) with D3 dispersion and Becke‐Johnson damping. Solvation used the SMD model with water as the solvent. All calculations were performed in Gaussian 16 Revision C.01. Analytical frequencies at the same level were used to obtain the thermal corrections. Free energies are the standard Gaussian values at 298.15 K and 1 atm. Relaxed dihedral scans used opt = modredundant over 36 steps of 10.0 degrees. Boronic acid pKa values were determined by isodesmic proton exchange. Phenylboronic acid was the reference acid at an experimental pKa of 8.8. The conjugate boronate was formed by hydroxide addition at the boron center. Binding free energies used catechol as the diol model. The reaction gives the cyclic boronate ester and two equivalents of water. Solvation free energies for P‐3+ and P‐6 were calculated in water and n‐octanol relative to the gas phase.

#### 4.5.15 Image Acquisition and Analysis

Strip images were captured under a handheld 365 nm source. A Nikon D5500 digital camera was used with the settings listed in the Supporting Information. All strip images were then analyzed in ImageJ. Test and control line intensities were measured over identical rectangular regions. A local background region of the same size was subtracted from each line. The T/C ratio was then formed from the corrected values. Strip panels in the main figures are cropped to the nitrocellulose region. Cropping was applied uniformly across every panel within a figure. The cropped panels come from the same original image files. The uncropped images are provided in the Supporting Information. All schemes and figures were created originally by the authors for this manuscript. No reprinted, adapted, or trademarked material is included anywhere.

#### 4.5.16 Probe and Test Line Screening Criteria

Six immunoprobes were screened against four test line chemistries. This gives twenty‐four combinations measured under identical conditions. The primary metric was the positive‐to‐negative ratio at 10 ng/mL antigen. The raw T/C ratio was not used as the primary metric. A combination with a higher T/C but a lower P/N was rejected. Such gains arise from nonspecific adsorption at the test line. Ties within experimental error were broken by the limit of detection.

#### 4.5.17 Statistical Analysis

Fluorescence images were analyzed in ImageJ as described above. No brightness, contrast, or gamma adjustment was applied at any stage. The reported T/C ratio is background corrected for both lines. No transformation or normalization was applied beyond this internal ratioing. No outliers were excluded from any dataset in this work. All measured replicates are included in every analysis reported. Data are presented as mean plus or minus standard deviation unless stated otherwise. Error bars represent the standard deviation of independent replicates. All analytical characterizations used *n* = 3 independent immunoprobe preparations. Each preparation was assayed on a separate test strip. Limits of detection were calculated as 3.3σ/k. Here, *σ* is the standard deviation of the blank measurements. The term *k* is the slope of the linear calibration range. Limits of detection used *n* = 3 replicate calibration series. Calibration curves were fitted by linear least squares regression. The coefficient of determination is reported for each fit. The clinical cohort comprised twenty‐eight subjects in total. Each sample was measured once by the LFA platform. Each sample was measured once by the reference method. Agreement is reported as the absolute and percentage deviation from ECLIA. Sample sizes are stated in each figure legend. All image analysis was performed using ImageJ. All curve fitting and plotting were done using OriginPro 9.1.

## Author Contributions

**Pin‐Hao Wang**: investigation, validation, formal analysis, data curation. **Shunmuga Nathan Shunmuga Nainar**: investigation, validation, writing – original draft, formal analysis, data curation. **Bing‐Han Zhuang**: software, data curation. **Shun‐Mao Yang**: project administration, methodology. **Hong‐Cheu Lin**: project administration, conceptualization. **Yang‐Hsiang Chan**: conceptualization, supervision, project administration, writing – review and editing, methodology, resources.

## Funding

The authors thank National Science and Technology Council (Grant No. 113‐2628‐M‐A49‐009‐MY3).

## Conflicts of Interest

The authors declare no conflicts of interest.

## Supporting information




**Supporting File 1**: adhm71815‐sup‐0001‐SuppMat.pdf.

**Supporting File 2**: adhm71815‐sup‐0002‐VideoS1.mov.

**Supporting File 3**: adhm71815‐sup‐0003‐VideoS2.mov.

## Data Availability

The main data supporting the findings of this study are available within the article, as well as the Supporting Information file. Should any raw data files be needed in another format, they are available from the corresponding author upon reasonable request.

## References

[1] J. Horgan , *Justinian's Plague (541‐542 CE)* In 2014; Updated December 26.

[2] WHO Coronavirus (COVID‐19) Data 2023, https://data.who.int/dashboards/covid19/more‐resources (accessed 2026 14 January).

[3] Y. Hayashi , R. Matsuda , T. Maitani , et al., “Precision, Limit of Detection and Range of Quantitation in Competitive ELISA,” Analytical Chemistry 76, no. 5 (2004): 1295–1301, 10.1021/ac0302859. 14987084

[4] S. Petralia and S. Conoci , “PCR Technologies for Point of Care Testing: Progress and Perspectives,” ACS Sensors 2, no. 7 (2017): 876–891, 10.1021/acssensors.7b00299. 28750519

[5] N. Jiang , R. Ahmed , M. Damayantharan , B. Ünal , H. Butt , and A. K. Yetisen , “Lateral and Vertical Flow Assays for Point‐of‐Care Diagnostics,” Advanced Healthcare Materials 8, no. 14 (2019): 1900244, 10.1002/adhm.201900244. 31081270

[6] S. Shao , X. Wang , C. Sorial , X. Sun , and X. Xia , “Sensitive Colorimetric Lateral Flow Assays Enabled by Platinum‐Group Metal Nanoparticles With Peroxidase‐Like Activities,” Advanced Healthcare Materials 14, no. 8 (2025): 2401677, 10.1002/adhm.202401677. PMC11799360 39108051

[7] P. Chowdhury , R. Lawrance , Z.‐Y. Lu , H.‐C. Lin , and Y.‐H. Chan , “Recent Progress in Dual/Multi‐Modal Detection Modes for Improving Sensitivity and Specificity of Lateral Flow Immunoassays Applied for Point‐of‐Care Diagnostics,” TrAc Trends in Analytical Chemistry 177 (2024): 117798, 10.1016/j.trac.2024.117798.

[8] S. Liu , Y. Liao , R. Shu , et al., “Evaluation of the Multidimensional Enhanced Lateral Flow Immunoassay in Point‐of‐Care Nanosensors,” ACS Nano 18, no. 40 (2024): 27167–27205, 10.1021/acsnano.4c06564. 39311085

[9] I. P. Azzam and L. Sherych , Discontinue the Use of Antigen Testing in Skilled Nursing Facilities until Further Notice (Nevada Department of Health and Human Services, 2020).

[10] Y. Liu , L. Zhan , Z. Qin , J. Sackrison , and J. C. Bischof , “Ultrasensitive and Highly Specific Lateral Flow Assays for Point‐of‐Care Diagnosis,” ACS Nano 15, no. 3 (2021): 3593–3611, 10.1021/acsnano.0c10035. 33607867

[11] B. Lu , M. R. Smyth , and R. O'Kennedy , “Tutorial Review. Oriented Immobilization of Antibodies and Its Applications in Immunoassays and Immunosensors,” The Analyst 121, no. 3 (1996): 29R–32R, 10.1039/AN996210029R. 8729652

[12] Z.‐Y. Lu and Y.‐H. Chan , “The Importance of Antibody Orientation for Enhancing Sensitivity and Selectivity in Lateral Flow Immunoassays,” Sensors & Diagnostics 3, no. 10 (2024): 1613–1634, 10.1039/D4SD00206G.

[13] N. Tajima , M. Takai , and K. Ishihara , “Significance of Antibody Orientation Unraveled: Well‐Oriented Antibodies Recorded High Binding Affinity,” Analytical Chemistry 83, no. 6 (2011): 1969–1976, 10.1021/ac1026786. 21338074

[14] B. Saha , T. H. Evers , and M. W. J. Prins , “How Antibody Surface Coverage on Nanoparticles Determines the Activity and Kinetics of Antigen Capturing for Biosensing,” Analytical Chemistry 86, no. 16 (2014): 8158–8166, 10.1021/ac501536z. 25048623

[15] K. Tripathi and J. D. Driskell , “Quantifying Bound and Active Antibodies Conjugated to Gold Nanoparticles: A Comprehensive and Robust Approach to Evaluate Immobilization Chemistry,” ACS Omega 3, no. 7 (2018): 8253–8259, 10.1021/acsomega.8b00591. PMC6072236 30087938

[16] L. Breydo , A. E. Sales , T. Frege , M. C. Howell , B. Y. Zaslavsky , and V. N. Uversky , “Effects of Polymer Hydrophobicity on Protein Structure and Aggregation Kinetics in Crowded Milieu,” Biochemistry 54, no. 19 (2015): 2957–2966, 10.1021/acs.biochem.5b00116. 25919930

[17] D. Lou , L. Fan , Y. Cui , Y. Zhu , N. Gu , and Y. Zhang , “Fluorescent Nanoprobes With Oriented Modified Antibodies to Improve Lateral Flow Immunoassay of Cardiac Troponin I,” Analytical Chemistry 90, no. 11 (2018): 6502–6508, 10.1021/acs.analchem.7b05410. 29697255

[18] T. Ming , Y. Cheng , Y. Xing , et al., “Electrochemical Microfluidic Paper‐Based Aptasensor Platform Based on a Biotin–Streptavidin System for Label‐Free Detection of Biomarkers,” ACS Applied Materials & Interfaces 13, no. 39 (2021): 46317–46324, 10.1021/acsami.1c12716. 34546713

[19] P.‐C. Lin , S.‐H. Chen , K.‐Y. Wang , et al., “Fabrication of Oriented Antibody‐Conjugated Magnetic Nanoprobes and Their Immunoaffinity Application,” Analytical Chemistry 81, no. 21 (2009): 8774–8782, 10.1021/ac9012122. 19874051

[20] Q. Yang , W. Zuo , J. Song , et al., “Dual‐Enhanced Lateral Flow Immunoassay: Synergizing Oriented Antibody Anchoring and Nanozyme Catalytic Amplification for Ultrasensitive Cancer Detection,” Analytical Chemistry 97, no. 31 (2025): 16942–16949, 10.1021/acs.analchem.5c02403. 40742195

[21] S. J. Walsh , J. D. Bargh , F. M. Dannheim , et al., “Site‐Selective Modification Strategies in Antibody–Drug Conjugates,” Chemical Society Reviews 50, no. 2 (2021): 1305–1353, 10.1039/D0CS00310G. 33290462

[22] C. J. Sadler , A. Creamer , K. A. Giang , et al., “Adding a Twist to Lateral Flow Immunoassays: A Direct Replacement of Antibodies With Helical Affibodies, From Selection to Application,” Journal of the American Chemical Society 147, no. 14 (2025): 11925–11940, 10.1021/jacs.4c17452. PMC11987028 40135773

[23] C.‐C. Fang , C.‐C. Chou , Y.‐Q. Yang , T. Wei‐Kai , Y.‐T. Wang , and Y.‐H. Chan , “Multiplexed Detection of Tumor Markers With Multicolor Polymer Dot‐Based Immunochromatography Test Strip,” Analytical Chemistry 90, no. 3 (2018): 2134–2140, 10.1021/acs.analchem.7b04411. 29286241

[24] M.‐W. Wang , Z.‐M. Chen , Y.‐C. Hsieh , et al., “Trimodal Multiplexed Lateral Flow Test Strips Assisted With a Portable Microfluidic Centrifugation Device,” Analytical Chemistry 96, no. 38 (2024): 15238–15247, 10.1021/acs.analchem.4c02432. PMC11428082 39276068

[25] Y.‐C. Yang , M.‐H. Liu , S.‐M. Yang , and Y.‐H. Chan , “Bimodal Multiplexed Detection of Tumor Markers in Non‐Small Cell Lung Cancer With Polymer Dot‐Based Immunoassay,” ACS Sensors 6, no. 11 (2021): 4255–4264, 10.1021/acssensors.1c02025. 34788538

[26] Y.‐C. Chen , Y.‐H. Syu , J.‐Y. Huang , C.‐Y. Lin , and Y.‐H. Chan , “Hybrid Polymer Dot‐magnetic Nanoparticle Based Immunoassay for Dual‐mode Multiplexed Detection of Two Mycotoxins,” Chemical Communications 59, no. 66 (2023): 9968–9971, 10.1039/D3CC02586A. 37501643

[27] Y.‐Q. Yang , Y.‐C. Yang , M.‐H. Liu , and Y.‐H. Chan , “FRET‐Created Traffic Light Immunoassay Based on Polymer Dots for PSA Detection,” Analytical Chemistry 92, no. 1 (2020): 1493–1501, 10.1021/acs.analchem.9b04747. 31815438

[28] P.‐Y. You , F.‐C. Li , M.‐H. Liu , and Y.‐H. Chan , “Colorimetric and Fluorescent Dual‐Mode Immunoassay Based on Plasmon‐Enhanced Fluorescence of Polymer Dots for Detection of PSA in Whole Blood,” ACS Applied Materials & Interfaces 11, no. 10 (2019): 9841–9849, 10.1021/acsami.9b00204. 30784256

[29] T. Mahmoudi , B. Shirdel , B. Mansoori , and B. Baradaran , “Dual Sensitivity Enhancement in Gold nanoparticle‐based lateral flow immunoassay for visual detection of carcinoembryonic antigen,” Analytical Science Advances 1, no. 3 (2020): 161–172, 10.1002/ansa.202000023. PMC10989072 38716129

[30] J. Fox , D. V. B. Batchelor , H. Roberts , et al., “Gold Nanotapes and Nanopinecones in a Quantitative Lateral Flow Assay for the Cancer Biomarker Carcinoembryonic Antigen,” ACS Applied Nano Materials 6, no. 19 (2023): 17769–17777, 10.1021/acsanm.3c03053. PMC10580237 37854850

[31] N. G. Welch , J. A. Scoble , B. W. Muir , and P. J. Pigram , “Orientation and Characterization of Immobilized Antibodies for Improved Immunoassays (Review),” Biointerphases 12, no. 2 (2017): 02D301, 10.1116/1.4978435. 28301944

[32] S. Gao , J. M. Guisán , and J. Rocha‐Martin , “Oriented immobilization of antibodies Onto sensing platforms—A critical review,” Analytica Chimica Acta 1189 (2022): 338907, 10.1016/j.aca.2021.338907. 34815045

[33] E. Beitello , K. Osei , T. Kobulnicky , F. Breausche , J. A. Friesen , and J. D. Driskell , “Oriented Surface Immobilization of Antibodies Using Enzyme‐Mediated Site‐Specific Biotinylation for Enhanced Antigen‐Binding Capacity,” Langmuir 41, no. 16 (2025): 10576–10585, 10.1021/acs.langmuir.5c00656. PMC12044683 40253628

[34] A. K. Trilling , J. Beekwilder , and H. Zuilhof , “Antibody Orientation on Biosensor Surfaces: A Minireview,” The Analyst 138, no. 6 (2013): 1619–1627, 10.1039/C2AN36787D. 23337971

[35] J.‐C. Yang , Y.‐C. Ho , and Y.‐H. Chan , “Ultrabright Fluorescent Polymer Dots With Thermochromic Characteristics for Full‐Color Security Marking,” ACS Applied Materials & Interfaces 11, no. 32 (2019): 29341–29349, 10.1021/acsami.9b10393. 31315396

[36] X. Bai , K. Wang , L. Chen , J. Zhou , and J. Wang , “Semiconducting Polymer Dots as Fluorescent Probes for in Vitro Biosensing,” Journal of Materials Chemistry B 10, no. 33 (2022): 6248–6262, 10.1039/D2TB01385A. 35971822

[37] J. Ferlay , M. Ervik , M. Laversanne , et al., “Global Cancer Observatory: Cancer Today,” *International Agency for Research on Cancer: Lyon* (2024), https://gco.iarc.who.int/ (accessed February 25, 2026).

[38] G. Springsteen and B. Wang , “A Detailed Examination of Boronic Acid–Diol Complexation,” Tetrahedron 58, no. 26 (2002): 5291–5300, 10.1016/S0040-4020(02)00489-1.

[39] J. Yan , G. Springsteen , S. Deeter , and B. Wang , “The relationship Among pKa, pH, and Binding Constants in the Interactions Between boronic acids and diols—it is not as Simple as it Appears,” Tetrahedron 60, no. 49 (2004): 11205–11209, 10.1016/j.tet.2004.08.051.

[40] H. Otsuka , E. Uchimura , H. Koshino , T. Okano , and K. Kataoka , “Anomalous Binding Profile of Phenylboronic Acid With N‐Acetylneuraminic Acid (Neu5Ac) in Aqueous Solution With Varying pH,” Journal of the American Chemical Society 125, no. 12 (2003): 3493–3502, 10.1021/ja021303r. 12643711

[41] Y.‐H. Chan , C. Wu , F. Ye , Y. Jin , P. B. Smith , and D. T. Chiu , “Development of Ultrabright Semiconducting Polymer Dots for Ratiometric pH Sensing,” Analytical Chemistry 83, no. 4 (2011): 1448–1455, 10.1021/ac103140x. PMC3039106 21244093

[42] S. C. Low , R. Shaimi , Y. Thandaithabany , J. K. Lim , A. L. Ahmad , and A. Ismail , “Electrophoretic Interactions Between Nitrocellulose Membranes and Proteins: Biointerface Analysis and Protein Adhesion Properties,” Colloids and Surfaces B: Biointerfaces 110 (2013): 248–253, 10.1016/j.colsurfb.2013.05.001. 23732801

[43] M.‐C. Chung , Y.‐Q. Liu , B.‐L. Jian , et al., “Affinity‐Switchable Interaction of Biotin and Streptavidin for the Signal‐ON Detection of Small Molecules,” ACS Sensors 8, no. 11 (2023): 4226–4232, 10.1021/acssensors.3c01572. 37871282

[44] J. Du , C. Cao , Z. Xue , et al., “AI‐Enhanced Lateral Flow Assay Enables 3‐Minute Quantitative Detection With Laboratory‐Grade Accuracy,” Analytical Chemistry 97, no. 43 (2025): 24196–24208, 10.1021/acs.analchem.5c05108. 41124618

[45] G.‐R. Han , A. Goncharov , M. Eryilmaz , et al., “Deep Learning‐Enhanced Paper‐Based Vertical Flow Assay for High‐Sensitivity Troponin Detection Using Nanoparticle Amplification,” ACS Nano 18, no. 41 (2024): 27933–27948, 10.1021/acsnano.4c05153. PMC11483942 39365271

[46] N. Zhang , S. Fu , P. Fu , and X. Xu , “DNA Tetrahedron Reformative Lateral Flow Assay for Improved Detection Sensitivity and Anti‐Interference,” Langmuir 41 (2025): 2036–2043, 10.1021/acs.langmuir.4c04663. 39792437

[47] C. A. C. Justo , V. Skouridou , P. Cools , G. Mulinganya , A. Ibáñez‐Escribano , and C. K. O'Sullivan , “Biotin/Avidin‐free Sandwich Aptamer‐based Lateral Flow Assay (ALFA) for the Diagnosis of Trichomonas Vaginalis,” Sensors & Diagnostics 4, no. 3 (2025): 216–228, 10.1039/d4sd00342j.

[48] Y. Nie , C. Shang , S. Fan ; et al., “A Multisite Bridging Mediated Lateral Flow Immunoassay for the Enhanced Detection of Mosquito‐borne Viruses,” Targetome 2, no. 2 (2026), 10.48130/targetome-0026-0016.

[49] Z. Wang , Y. Chen , Y. Wang , W. Li , X. Zhang , and S. Zhang , “One‐Pot Ligation‐Recombinase Polymerase Amplification‐Clustered Regularly Interspaced Short Palindromic Repeats/Cas12a‐Powered Trimode Lateral Flow Assay for Sensitive MicroRNA Detection,” Analytical Chemistry 98, no. 18 (2026): 13365–13376, 10.1021/acs.analchem.5c07208. 42046341

[50] C.‐P. Chen , Y.‐C. Huang , S.‐Y. Liou , P.‐J. Wu , S.‐Y. Kuo , and Y.‐H. Chan , “Near‐Infrared Fluorescent Semiconducting Polymer Dots With High Brightness and Pronounced Effect of Positioning Alkyl Chains on the Comonomers,” ACS Applied Materials & Interfaces 6, no. 23 (2014): 21585–21595, 10.1021/am506577r. 25394668

[51] H.‐Y. Liu , P.‐J. Wu , S.‐Y. Kuo , et al., “Quinoxaline‐Based Polymer Dots With Ultrabright Red to Near‐Infrared Fluorescence for in Vivo Biological Imaging,” Journal of the American Chemical Society 137, no. 32 (2015): 10420–10429, 10.1021/jacs.5b06710. 26255823

[52] P. Pracht , F. Bohle , and S. Grimme , “Automated Exploration of the Low‐energy Chemical Space With Fast Quantum Chemical Methods,” Physical Chemistry Chemical Physics 22, no. 14 (2020): 7169–7192, 10.1039/C9CP06869D. 32073075

[53] C. Bannwarth , S. Ehlert , and S. Grimme , “GFN2‐xTB─an Accurate and Broadly Parametrized Self‐Consistent Tight‐Binding Quantum Chemical Method With Multipole Electrostatics and Density‐Dependent Dispersion Contributions,” Journal of Chemical Theory and Computation 15, no. 3 (2019): 1652–1671, 10.1021/acs.jctc.8b01176. 30741547

