# Supplementary material for: Rational Control of Antibody Orientation for Enhancing Sensitivity and Specificity in Lateral Flow Immunoassays With Clinical Validation
Source: Adv Healthc Mater. 2026 Oct 9;15(41):e71815. doi: 10.1002/adhm.71815 (PMC13740947; doi:10.1002/adhm.71815)
Supplement: Supplementary file 1 [file ADHM-15-0-s002.pdf]

Rational Control of Antibody Orientation for Enhancing Sensitivity and Specificity in Lateral Flow Immunoassays with Clinical Validation

Pin-Hao Wang,[1†] Shunmuga Nathan Shunmuga Nainar,[2†] Bing-Han Zhuang,[1] Shun-Mao Yang,[3] Hong-Cheu Lin,*[2] and Yang-Hsiang Chan*[145]*

Mr. P.-H. Wang,[†] Mr. B.-H. Zhuang, and Prof. Y.-H. Chan

[1]Department of Applied Chemistry, National Yang Ming Chiao Tung University, Hsinchu, Taiwan 30010.

E-mail: yhchan@nycu.edu.tw

Mr. S.N.S. Nainar[†] and Prof. H.-C. Lin

[2]Department of Materials Science and Engineering, National Yang Ming Chiao Tung University, Hsinchu, Taiwan, 30010.

E-mail: linhc@nycu.edu.tw

Dr. S.-M. Yang

[3]Department of Surgery, National Taiwan University Hospital, Hsinchu Branch, Hsinchu, Taiwan 30010.

E-mail: mutayang@gmail.com

Prof. Y.-H. Chan

[4]Center for Emergent Functional Matter Science, National Yang Ming Chiao Tung University, Hsinchu, Taiwan 30010.

[5]Department of Medicinal and Applied Chemistry, Kaohsiung Medical University, Kaohsiung, Taiwan 80708.

[†]P.-H. Wang and S.N.S. Nainar contributed equally to this work.

Contents

Scheme S1. Synthesis of visibly emissive PFTC6FQ Pdots……………………………........(S4)

Figure S1. DLS and Zeta Potential of the P-1, P-2, P-4, and P-6 probes……………….........(S4)

Figure S2. Agarose Gel Electrophoresis……………………………………………….…….(S5)

Figure S3. Quantification of free Fc domains..………………………………………….........(S5)

Figure S4. Quantification of Fab region via BCA Analysis……...………………………......(S6)

Figure S5. Evaluation of P-6 antibody orientation through FRET….………………………….(S6)

Figure S6. Optimal probe loading in the strips……………………………………………….(S7)

Figure S7. Investigation of excitation and detection parameters……………………………..(S8)

Figure S8. Protocols for calculating T/C ratios………………………………………………(S9)

Figure S9. Quantitative comparison of CYFRA21-1 sensitivities..………………………….(S9)

Figure S10. Quantitative comparison of CEA sensitivities.…………………………..……..(S10)

Figure S11. Physicochemical matching and performance of the P-3+ control probe………(S11)

Figure S12. DFT analysis of the meta and para boronic acid linkers………………………(S12)

Figure S13. Uncropped strip images corresponding to Figure 3……………………………(S12)

Figure S14. Uncropped strip images corresponding to Figure 4……………………………(S13)

Figure S15. Uncropped strip images corresponding to Figure 5……………………………(S13)

Figure S16. Uncropped strip images corresponding to Figure 6…………………………....(S14)

Figure S17. Uncropped strip images corresponding to Figure 7……………………………(S14)

Figure S18. Uncropped strip images corresponding to Figure 8…………………………....(S15)

Figure S19. Matrix-matched calibration of CYFRA21-1 in human serum…………………(S16)

Figure S20. Computed pKa values and pH-dependent antibody loading…………………..(S16)

Table S1. Comparison table with other reported methods………………………….............(S17)

References…………………………………………………………………………….…….(S17)

ABBREVIATIONS

**CYFRA21-1**	Cytokeratin Fragment 21-1
**CEA**	Carcinoembryonic Antigen
**AFP**	Alpha Fetoprotein
**CA15-3**	Carbohydrate Antigen 15-3
**CA125**	Carbohydrate Antigen 125
**PSA**	Prostate Specific Antigen
**PEG**	Polyethylene Glycol
**FRET**	Förster Resonance Energy Transfer
**PPE**	Poly(p-phenylene ethynylene)
**FITC**	Fluorescein Isothiocyanate
**EDC**	1-Ethyl-3-(3-dimethylaminopropyl)carbodiimide
**NHS**	N-hydroxy succinimide
**BCA**	Bicinchoninic Acid
**LoD**	Limit of Detection
**SA**	Streptavidin

Scheme S1. Preparation and Surface Functionalization of PFTC6FQ Pdots.

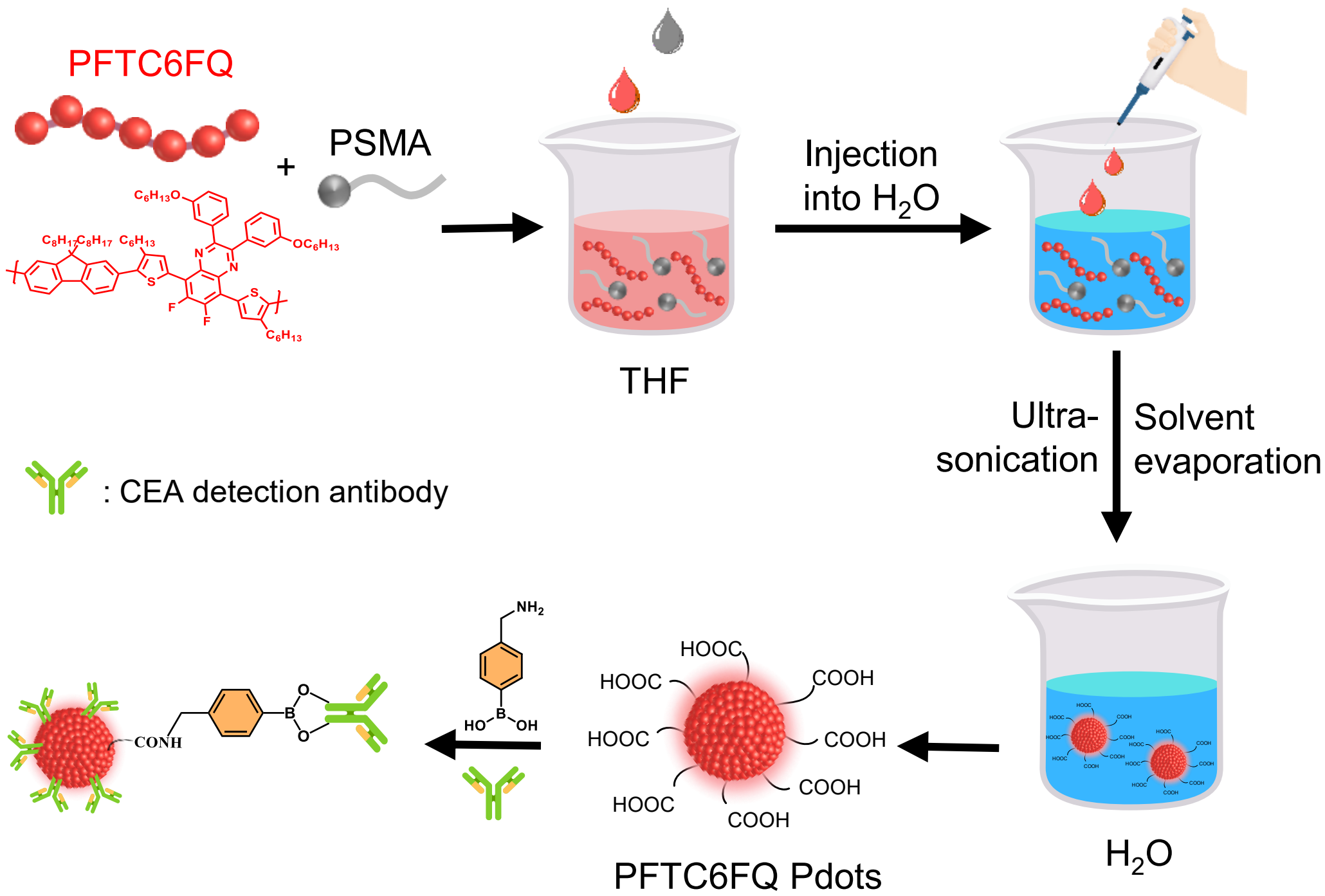

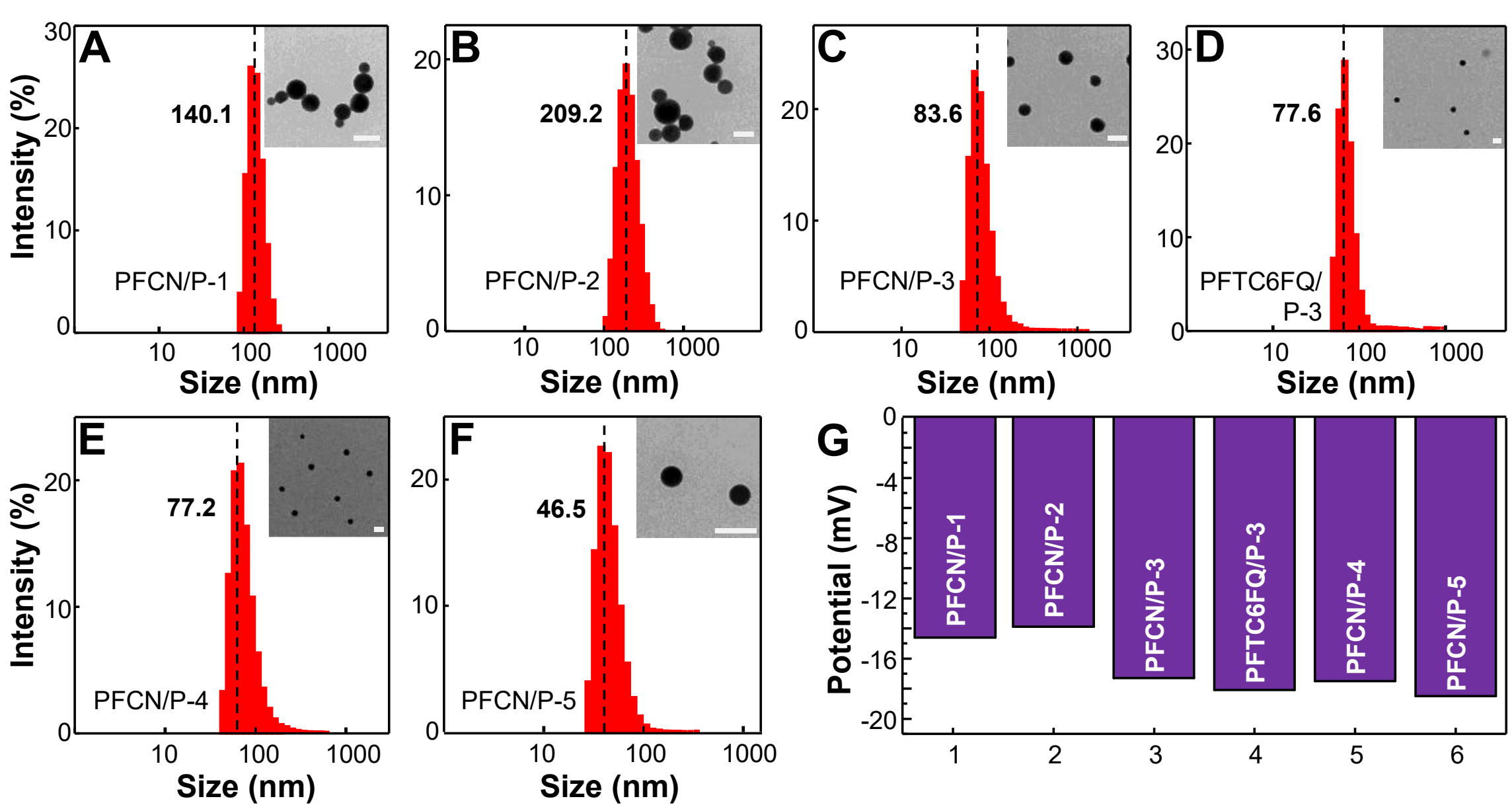

Figure S1. Dynamic light scattering (DLS) and zeta potential characterization of the synthesized immunoprobes. DLS data of (A) PFCN/P-1, (B) PFCN/P-2, (C) PFCN/P-3, (D) PFTC6FQ/P-3, (E) PFCN/P-4, and (F) PFCN/P-5, respectively. Insets are their corresponding TEM images. Scale bars are 100 nm. (G) Zeta potential values of the PFCN/PFTC6FQ-based immunoprobes 1-5.

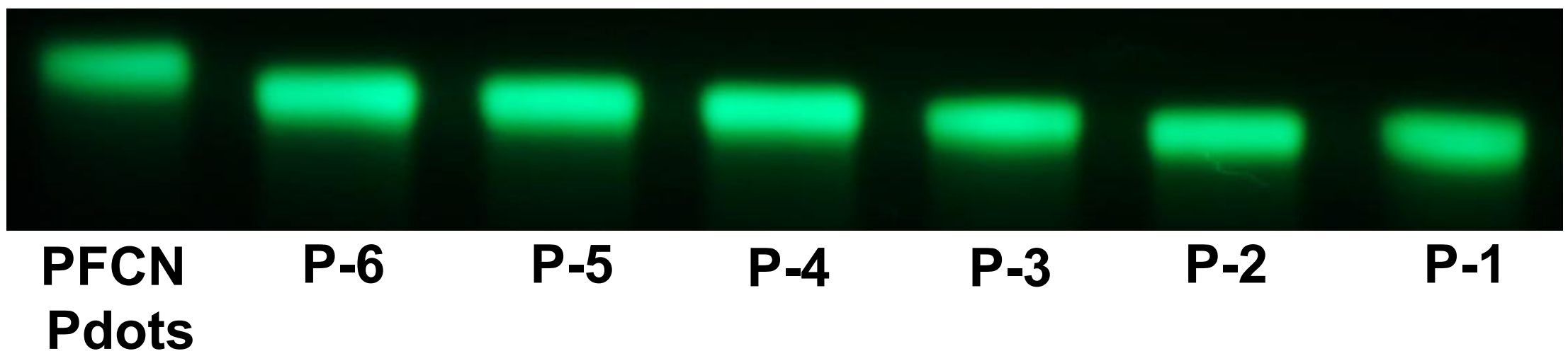

Figure S2. Agarose gel electrophoresis image under 365 nm UV illumination of bare PFCN Pdots and immunoprobes P-1 to P-6 with a bandpass filter of 525±25 nm.

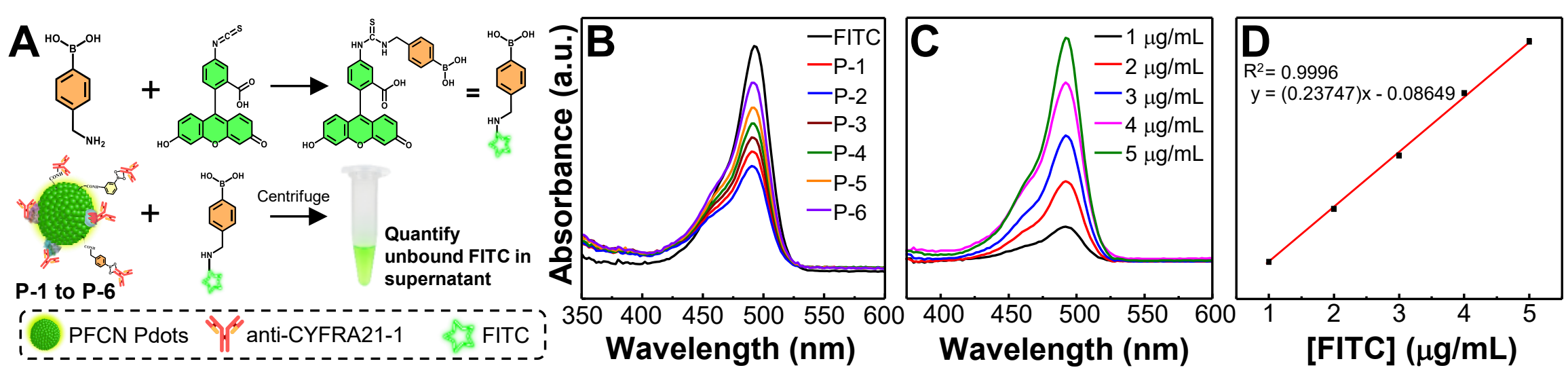

Figure S3. Quantification of free Fc domains using FITC–boronic acid labeling. (A) Schematic illustration of the assay. FITC-conjugated 4-(aminomethyl)phenylboronic acid selectively binds cis-diols on Fc-associated glycans that remain unengaged with the nanoparticle surface. After incubation with each immunoprobe (P-1 to P-6) and centrifugation, the unbound FITC reporter in the supernatant is quantified by UV-vis absorbance. (B) Absorbance spectra (350-600 nm) of supernatants after labeling, showing the characteristic FITC peak at 496 nm for each probe. (C) Calibration curve constructed from known FITC concentrations (1-5 μg/mL), enabling conversion of absorbance at 496 nm to free Fc content. (D) Linear relationship between absorbance and FITC concentration, validating quantitative analysis.

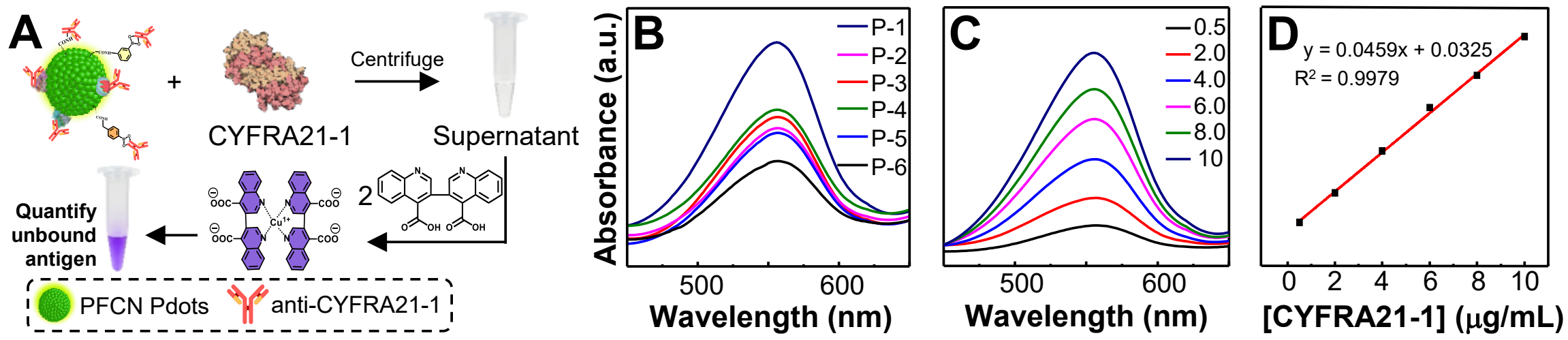

Figure S4. Evaluation of antigen-binding activity via BCA assay. (A) Schematic of the antigen-binding assay. Immunoprobes (P-1 to P-6) are incubated with a fixed concentration of CYFRA21-1 antigen (10 μg/mL). Following centrifugation, the unbound antigen in the supernatant is quantified by bicinchoninic acid (BCA) colorimetry, and the captured antigen is calculated by the difference. (B) Absorbance spectra corresponding to residual antigen concentrations in the supernatant for each probe. (C) BCA calibration curve (0.5-10 μg/mL CYFRA21-1), showing a concentration-dependent increase in absorbance. (D) Linear fit of the calibration data, confirming reliable quantification.

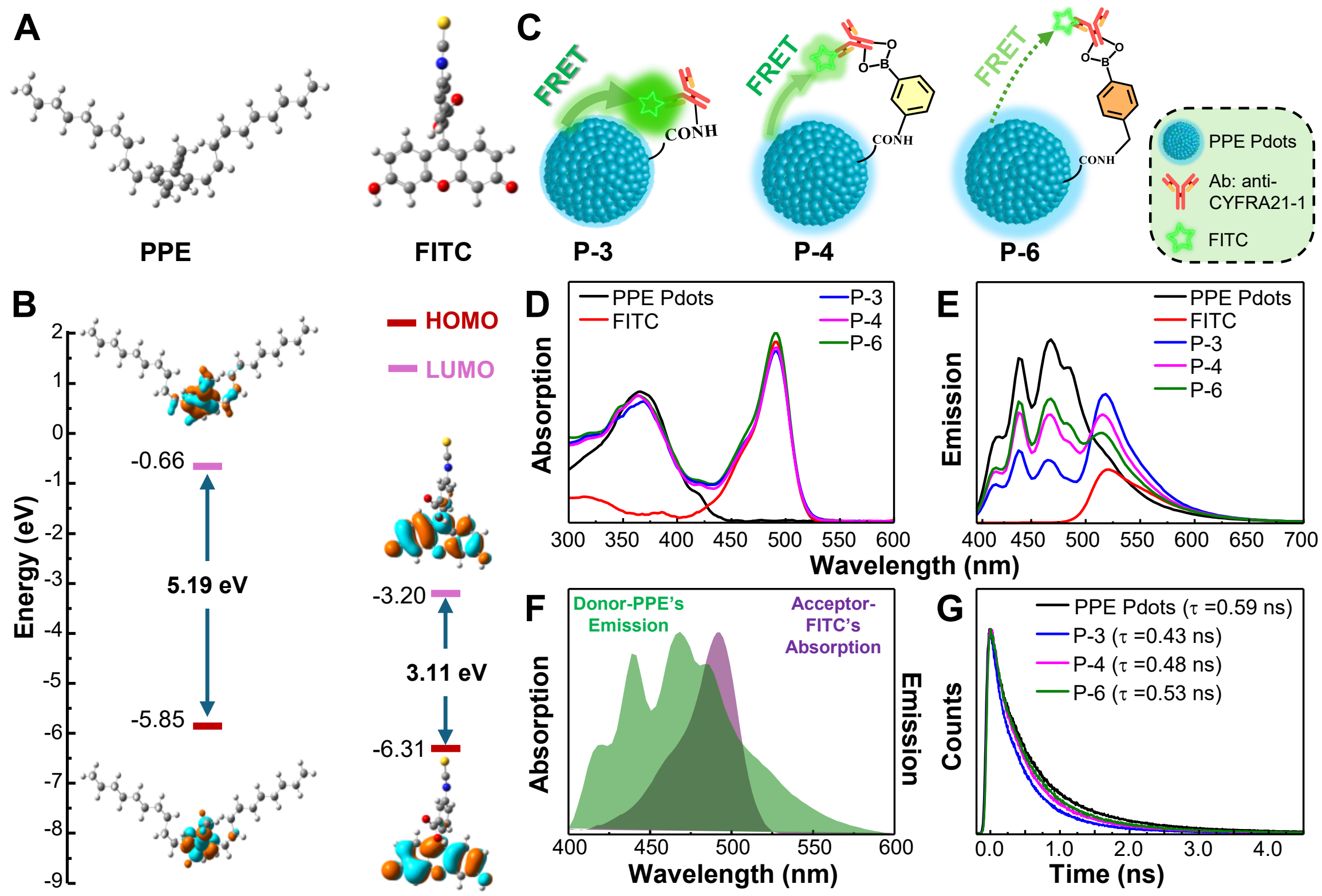

Figure S5. Evaluation of P-6 antibody orientation through FRET. (A) Optimized electronic structure of PPE and FITC, (B) Frontier Molecular Orbitals of the optimized structure of PPE and FITC, where the regions of positive potential are depicted in orange colour and the regions of negative potential are depicted in cyan colour. (C) Schematic illustrating FRET between PPE and FITC-coupled anti-CYFRA21-1. (D) Absorption, and (E) Emission spectra for the PPE Pdots, FITC, P-3, P-4, and P-6. (F) Spectral overlap between the donor's emission and acceptor's absorption. (G) Lifetime analysis showing donor quenching. The geometry optimization of the structure was calculated at B3LYP/6-31 G+(d,p)-SDD basis set with Gaussian 16W software suite.

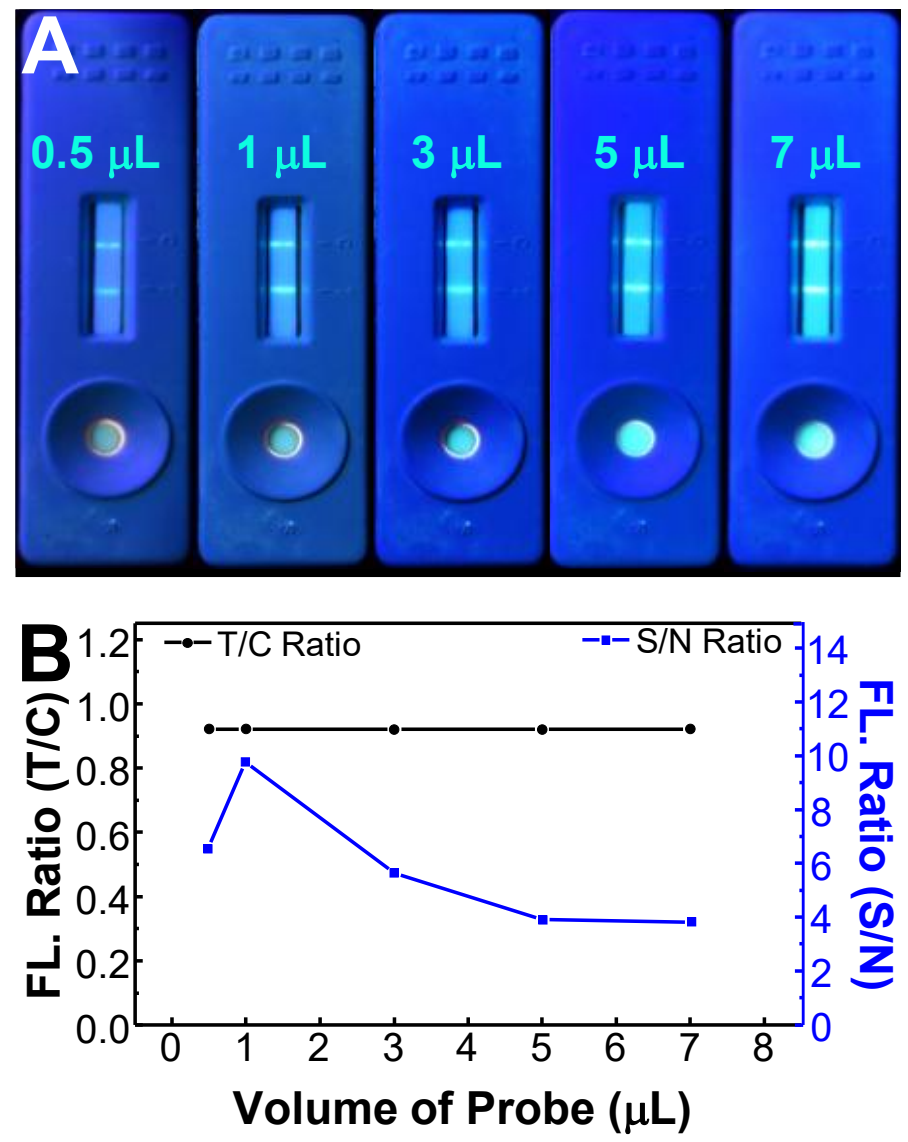

Figure S6. Optimization of probe loading volume for the P-6/biotin-SA LFA platform. (A) Fluorescence images of test strips under 365 nm UV excitation after application of 10 ng/mL CYFRA21-1 using PFCN/P-6 immunoprobes at five loading volumes: 0.5, 1, 3, 5, and 7 μL. (B) Corresponding fluorescence T/C ratio (left axis, black circles) and S/N ratio (right axis, blue squares) as a function of probe volume.

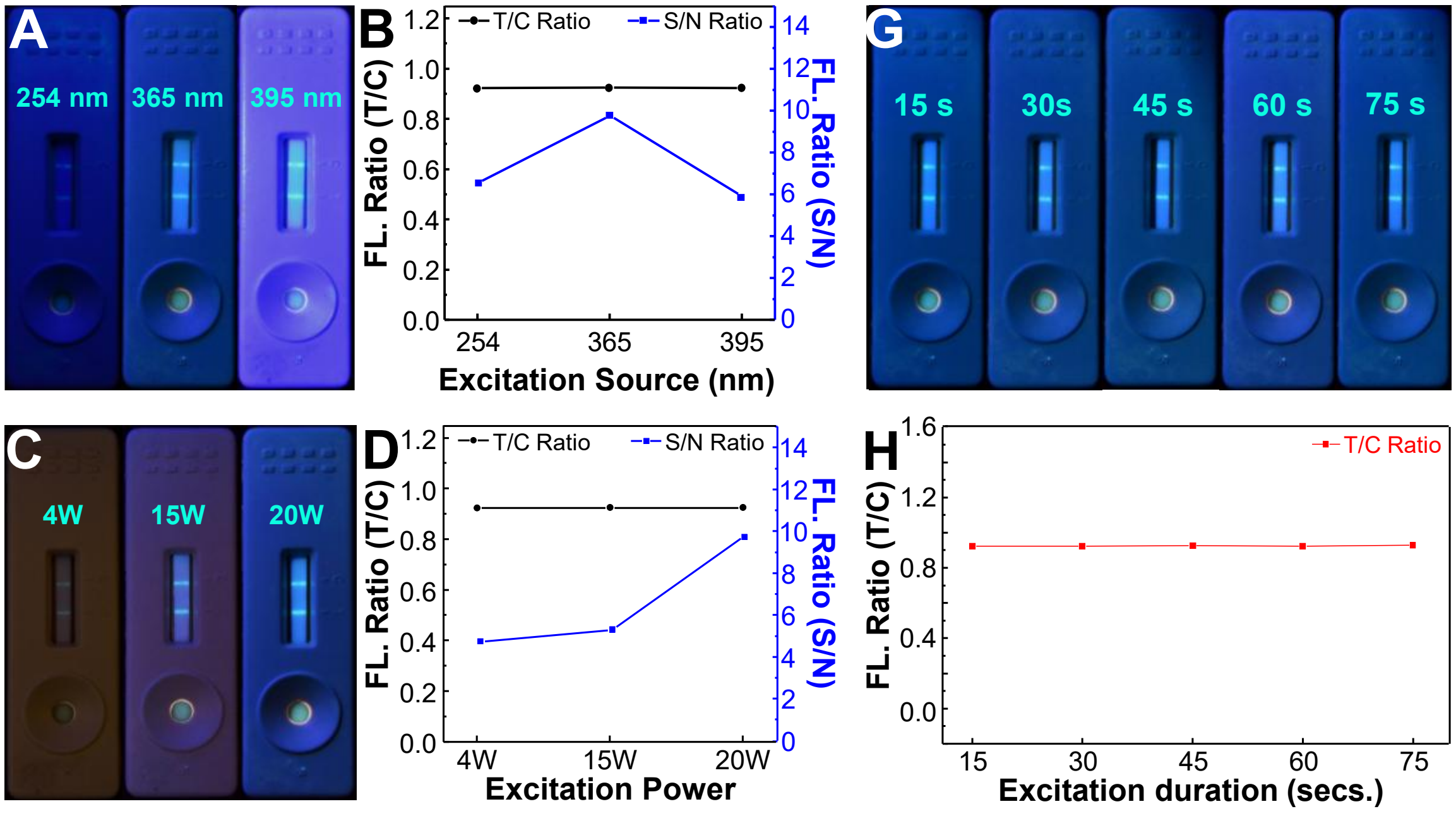

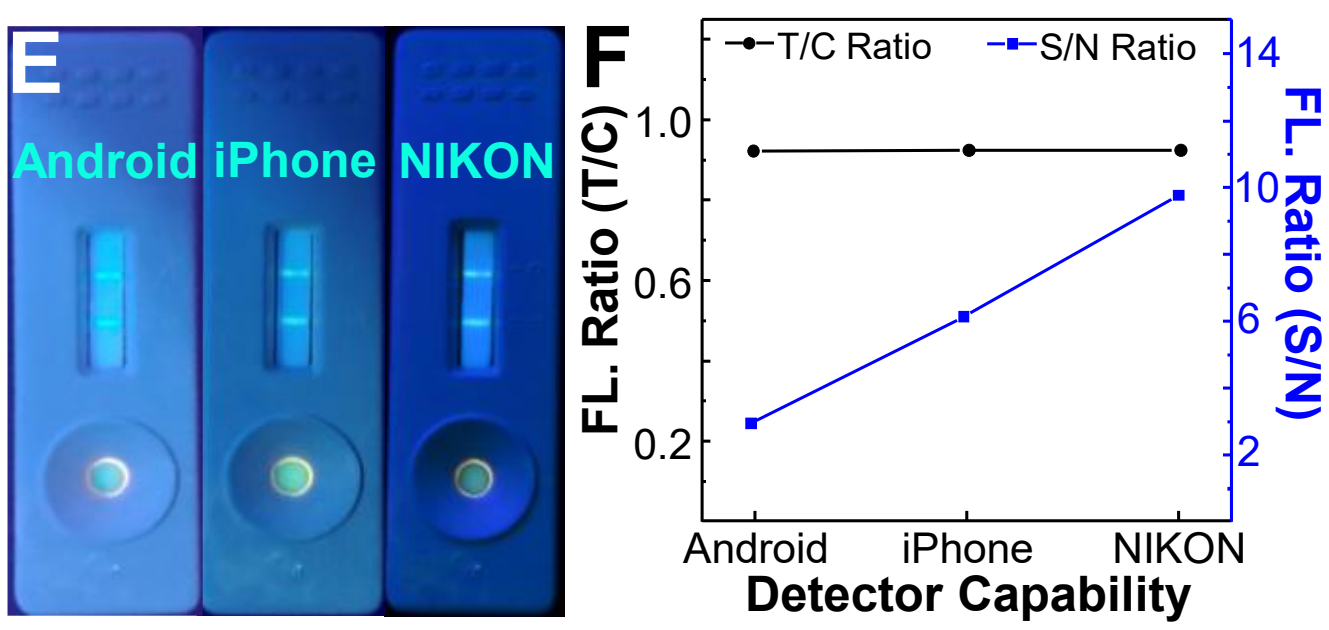

I

Parameter	Conditions	Optimal
Excitation source	254/365/395 nm	365 nm
Excitation power	4/15/20W	20W
Detector Capability	Android/ iPhone/ NIKON	NIKON
Excitation duration	15/30/45/60/75 s	15 s

Figure S7. Systematic investigation of imaging parameters for the fluorescence LFA readout. (A) Strip images under three excitation wavelengths: 254, 365, and 395 nm, and (B) corresponding T/C and S/N ratios. (C) Strip images using UV lamps of three power ratings: 4 W, 15 W, and 20 W, and (D) corresponding T/C and S/N ratios. (E) Strip images obtained with three different detectors: Android smartphone, iPhone, and NIKON D5500 digital camera, and (F) corresponding T/C and S/N ratios. (G) Strip images as a function of excitation duration: 15, 30, 45, 60, and 75 s, and (H) corresponding T/C ratios. (I) Summary table of evaluated conditions and identified optimal parameters. All strips were loaded with 10 ng/mL CYFRA21-1 using the P-6/biotin-SA configuration. The T/C ratio is unchanged across the three detectors tested in panels (E) and (F). The ratio is internally referenced within a single image frame. Both lines are imaged through the same optics and illumination. Detector gain and exposure, therefore, cancel in the ratio. Only the signal-to-noise ratio differs between the detectors. The digital camera was selected for its higher signal-to-noise ratio. Its acquisition settings are also fixed and fully reportable. Another laboratory can therefore reproduce these imaging conditions exactly.

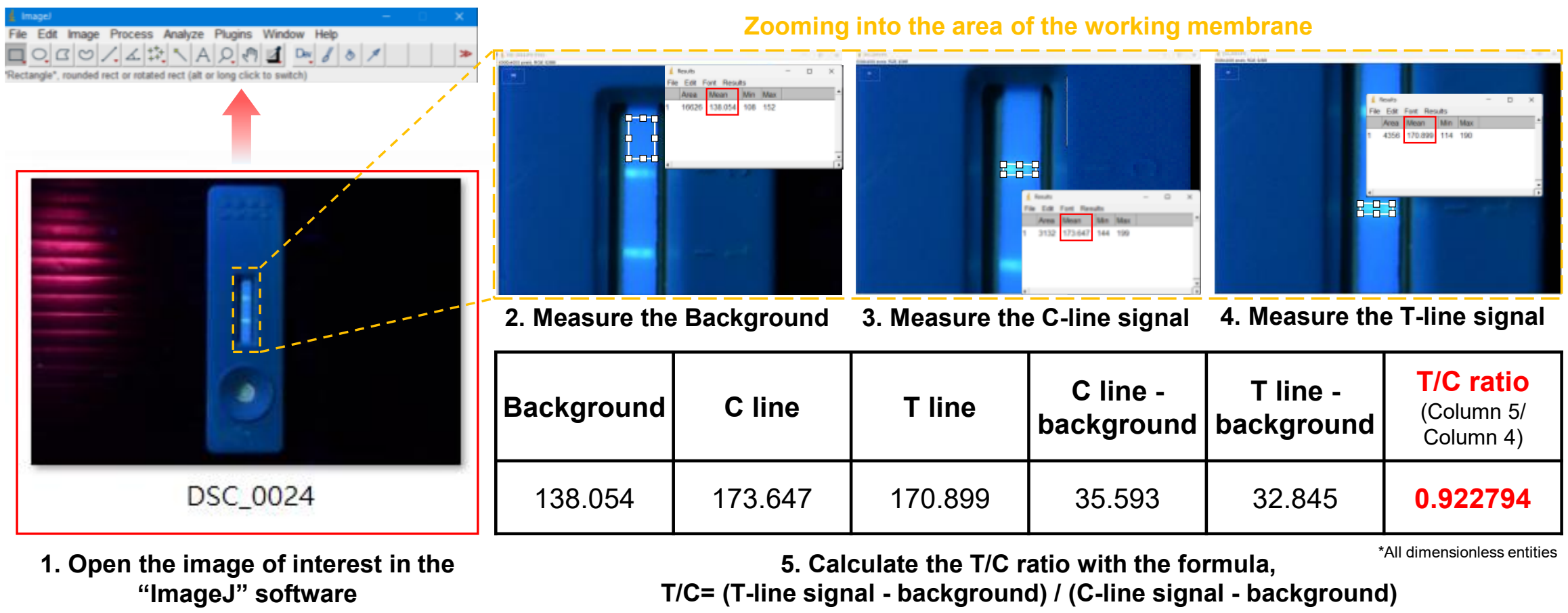

Figure S8. Step-by-step protocol for background-corrected T/C ratio calculation using ImageJ software.

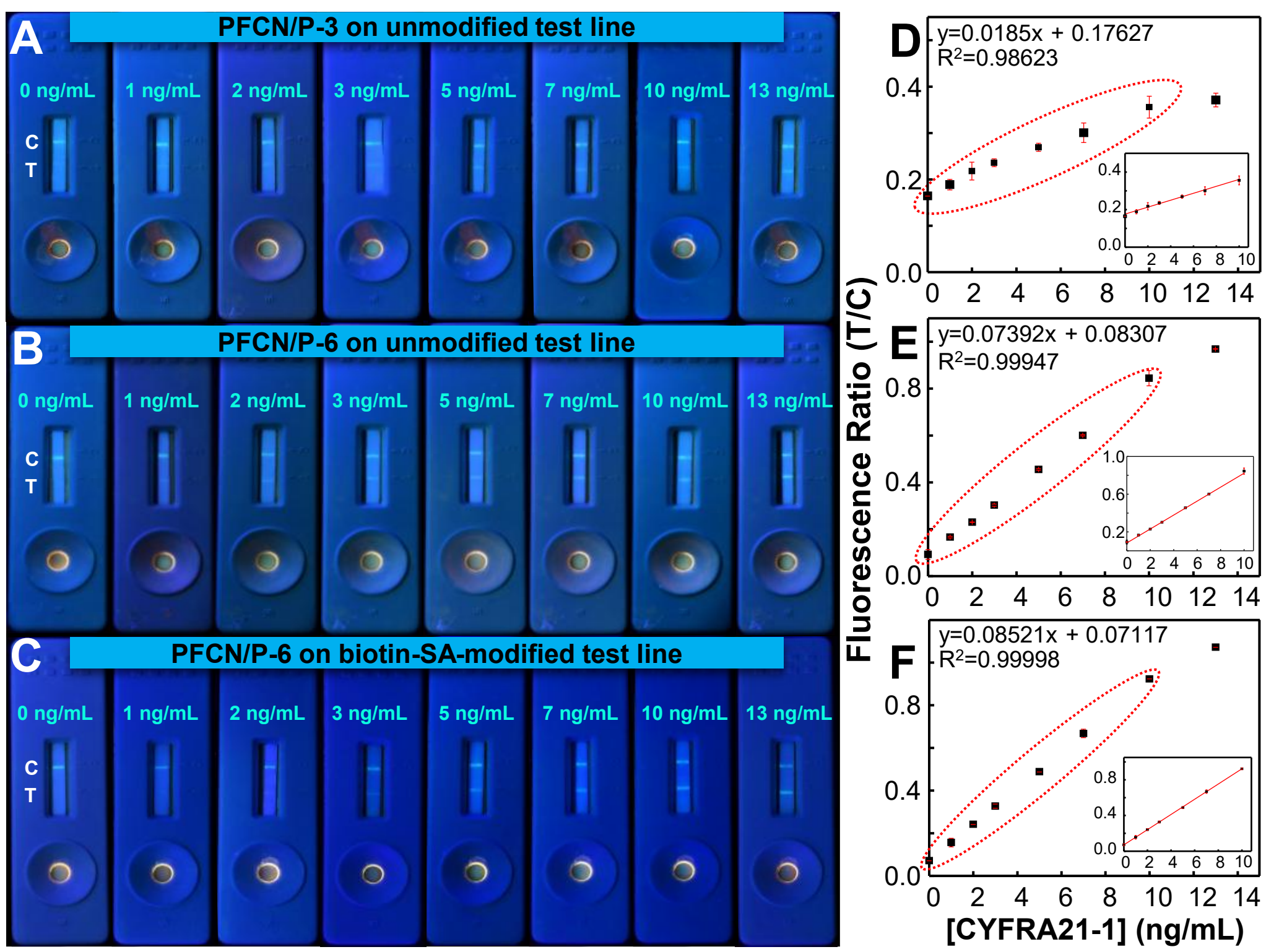

Figure S9. Quantitative comparison of fluorescence-based sensitivities for CYFRA21-1 detection. Fluorescence images of test strips under 365 nm excitation with CYFRA21-1 concentrations of 0-13 ng/mL using (A) PFCN/P-3 on unmodified test lines, (B) PFCN/P-6 on unmodified test lines, and (C) PFCN/P-6 on biotin-SA-modified test lines. (D-F) Corresponding calibration plots and dynamic ranges for A-C, respectively.

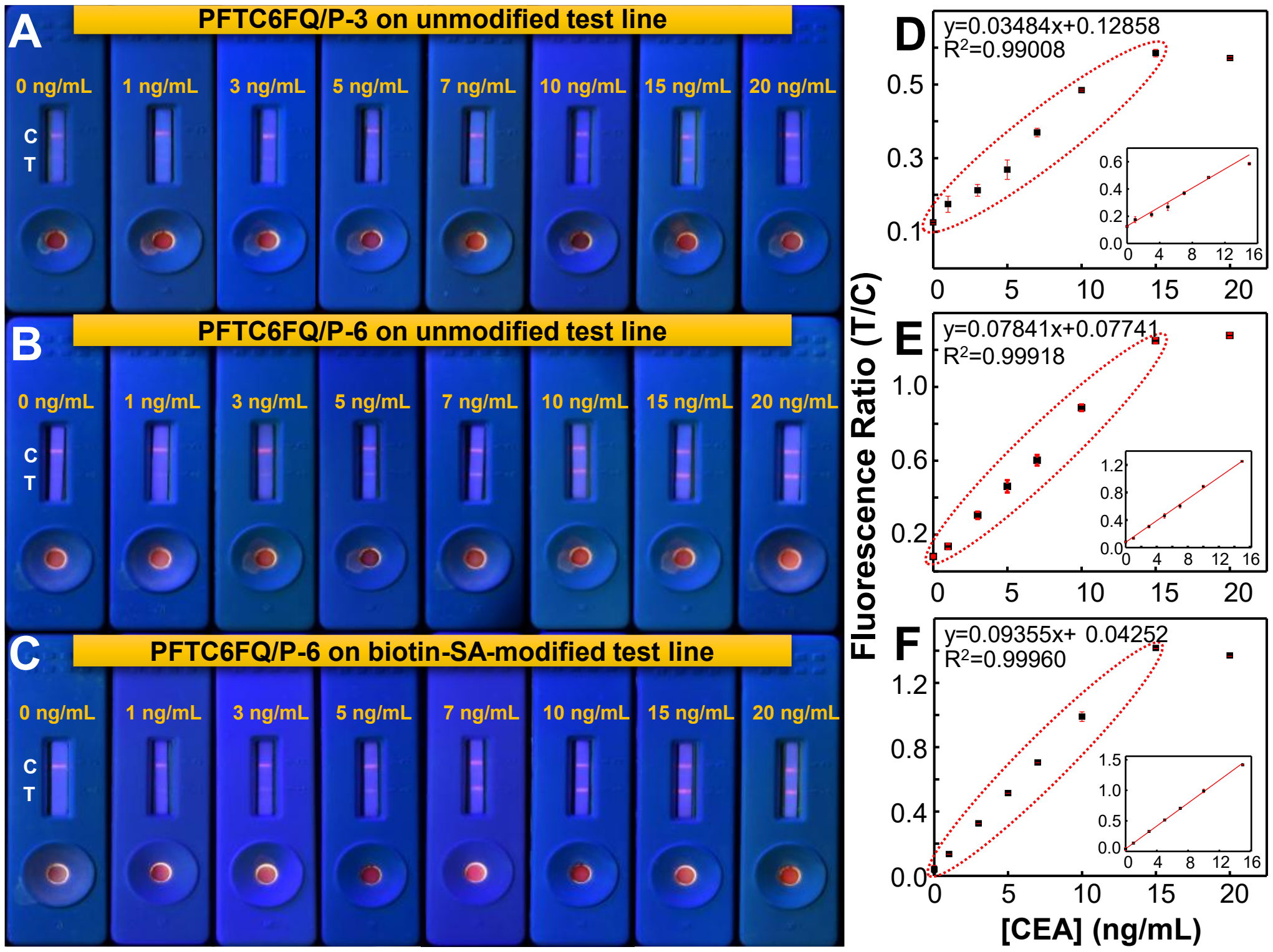

Figure S10. Quantitative comparison of fluorescence-based sensitivities for CEA detection. Fluorescence images of test strips under 365 nm excitation with CEA concentrations of 0-20 ng/mL using (A) PFTC6FQ/P-3 on unmodified test lines, (B) PFTC6FQ/P-6 on unmodified test lines, and (C) PFTC6FQ/P-6 on biotin-SA-modified test lines. (D-F) Corresponding calibration plots and dynamic ranges for A-C, respectively.

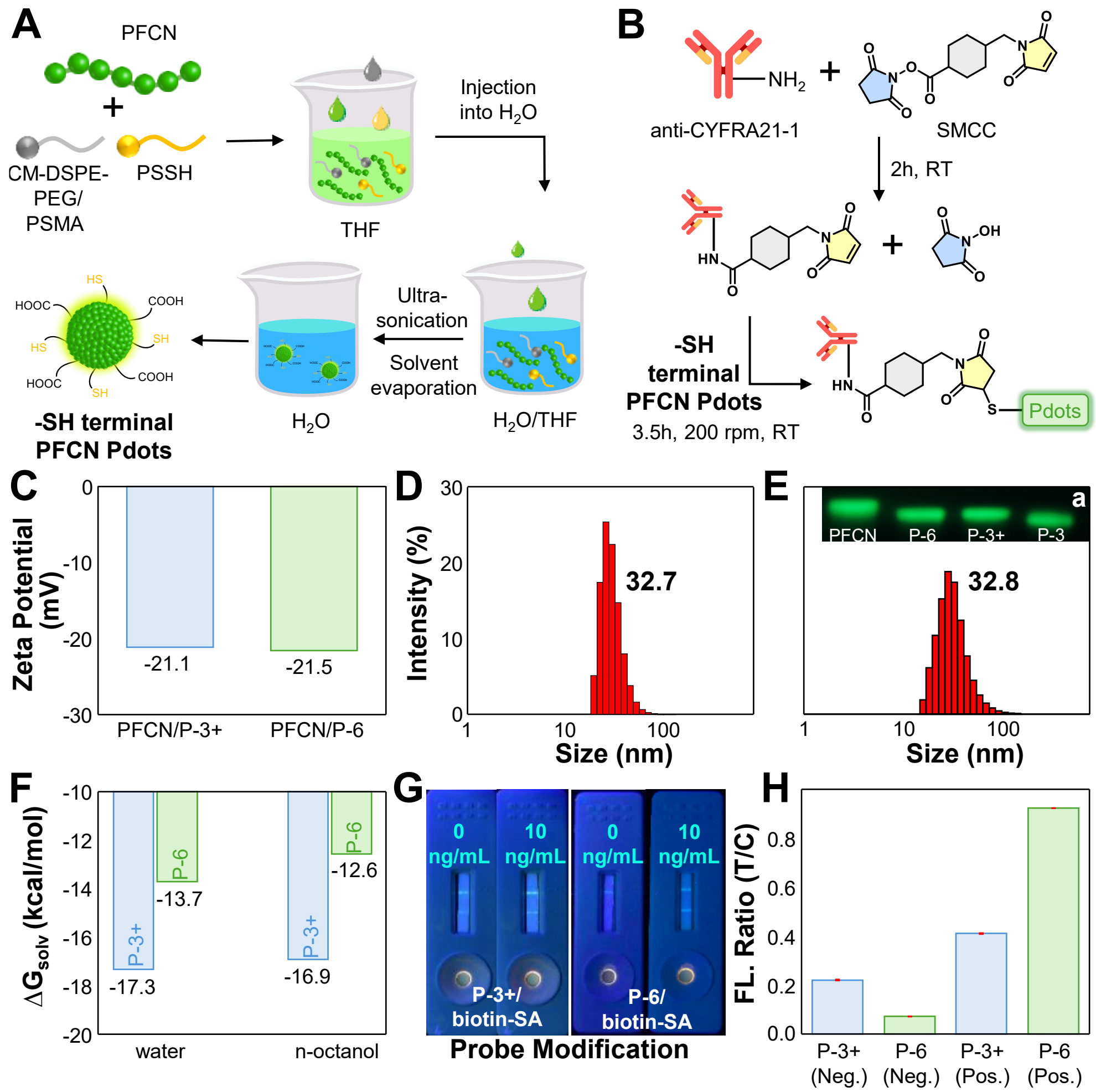

Figure S11. Design and performance of the P-3+ control probe. (A) Preparation of thiol-terminated PFCN Pdots using PSSH with CM-DSPE-PEG and PSMA. (B) Activation of anti-CYFRA21-1 with SMCC for 2 h at room temperature. The activated antibody is then coupled to the Pdot thiols for 3.5 h at 200 rpm. (C) Zeta potentials of PFCN/P-3+ and PFCN/P-6, measured at -21.1 and -21.5 mV. (D, E) Hydrodynamic diameters of the same two probes, at 32.7 and 32.8 nm. The inset in (E) shows the agarose gel image of bare PFCN Pdots and of P-6, P-3+, and P-3. (F) Computed solvation free energies of the two linkers in water and n-octanol. (G) Fluorescence test strip images of P-3+ and P-6 probes under 0 and 10 ng/mL of CYFRA21-1 antigen. (H) Their corresponding T/C ratios. Data in (C), (D), (E), and (H) are mean ± standard deviation of n = 3 independent preparations.

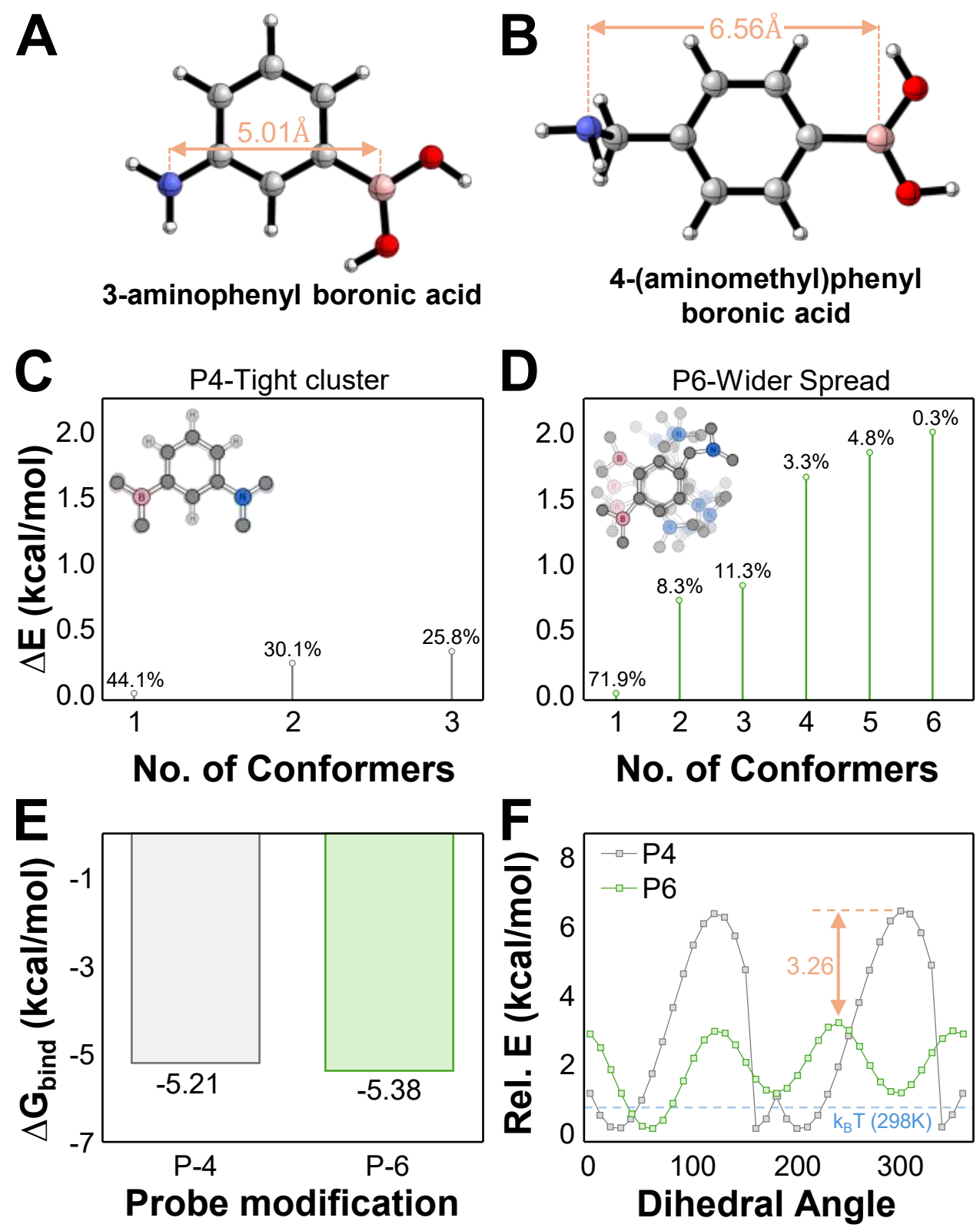

Figure S12. DFT analysis of the meta and para-substituted boronic acid linkers. (A) Optimized structure of 3-aminophenylboronic acid, showing a nitrogen to boron distance of 5.01 Å. (B) Optimized structure of 4-(aminomethyl)phenylboronic acid, showing a distance of 6.56 Å. (C) Conformer ensemble of the P-4 linker. Three rotamers account for 44.1 percent, 30.1 percent, and 25.8 percent of the population, respectively. These three states span only 0.32 kcal/mol in total. (D) Conformer ensemble of the P-6 linker. Six conformers span 1.99 kcal/mol with a dominant basin at 71.9 percent. (E) Binding free energies for boronate ester formation with catechol, at -5.21 and -5.38 kcal/mol. (F) Relaxed dihedral scans for P-4 and P-6 showing the rotational barriers of the corresponding immunoprobes.

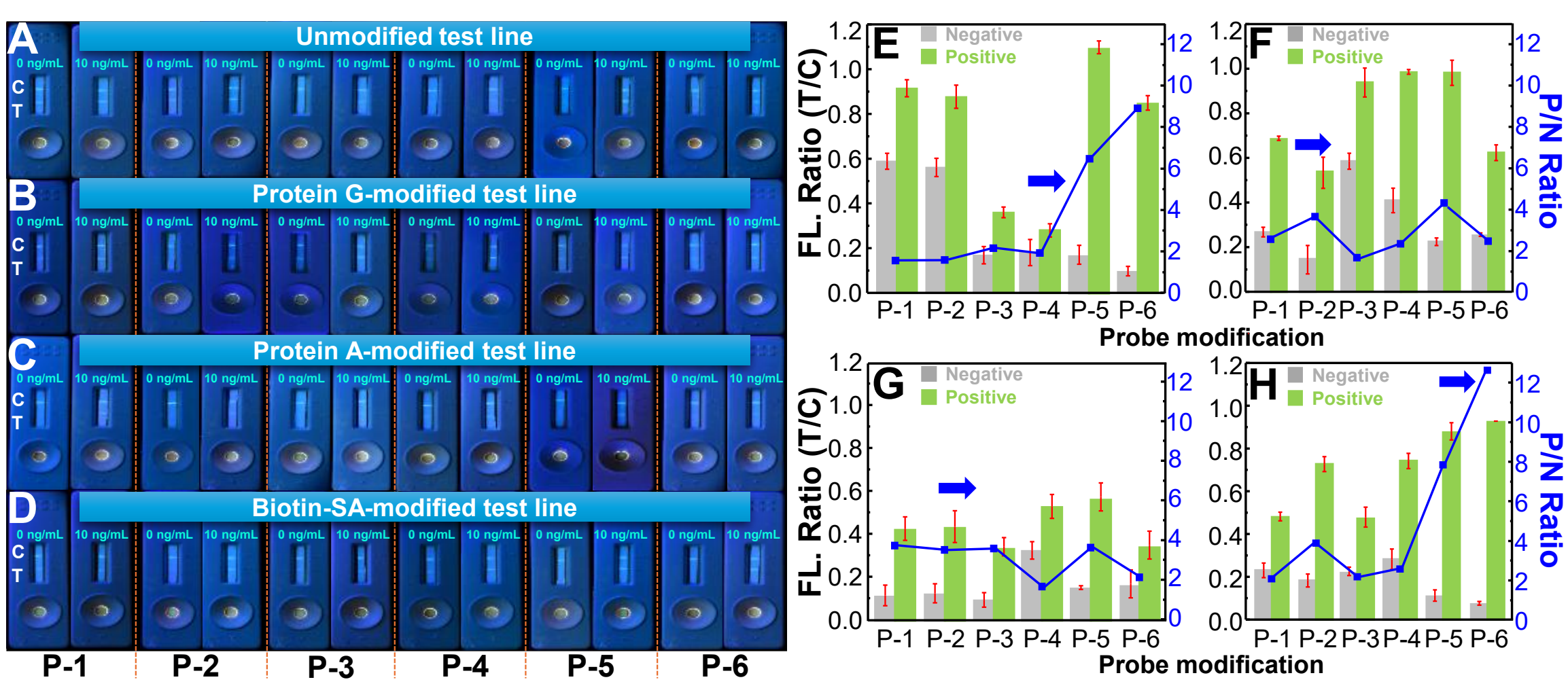

Figure S13. Uncropped test strips images of Figure 3.

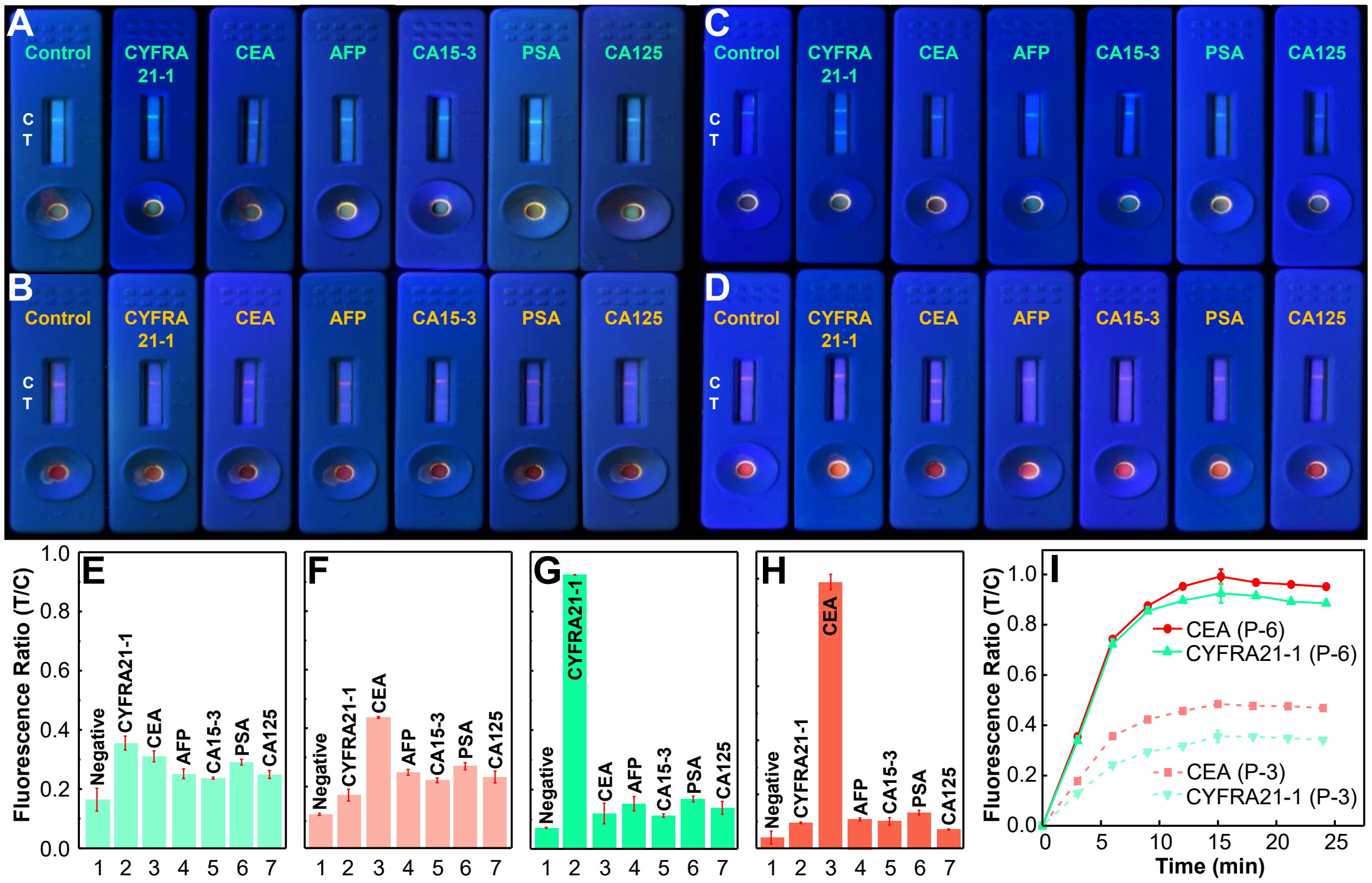

Figure S14. Uncropped test strips images of Figure 4.

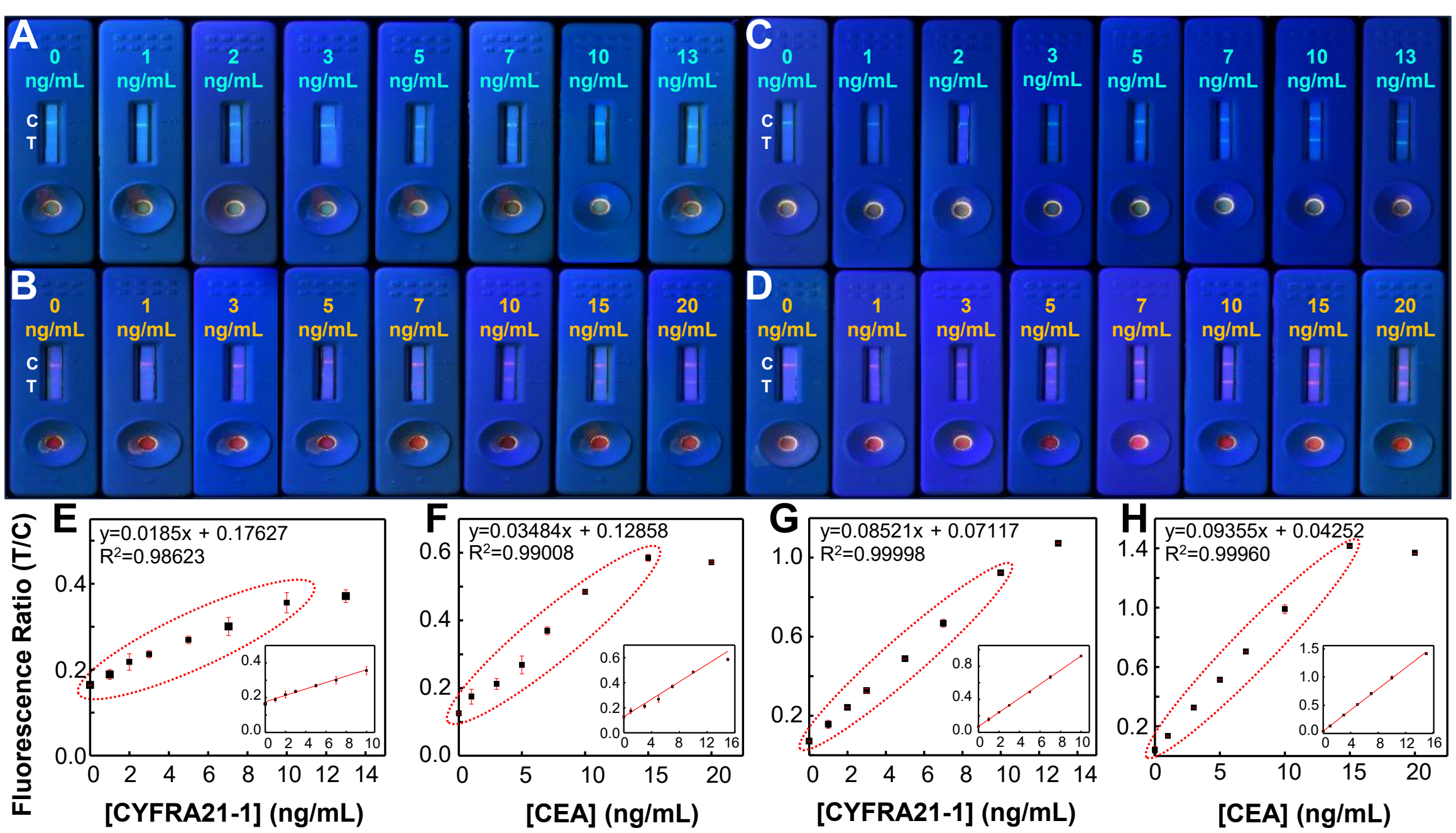

Figure S15. Uncropped test strips images of Figure 5.

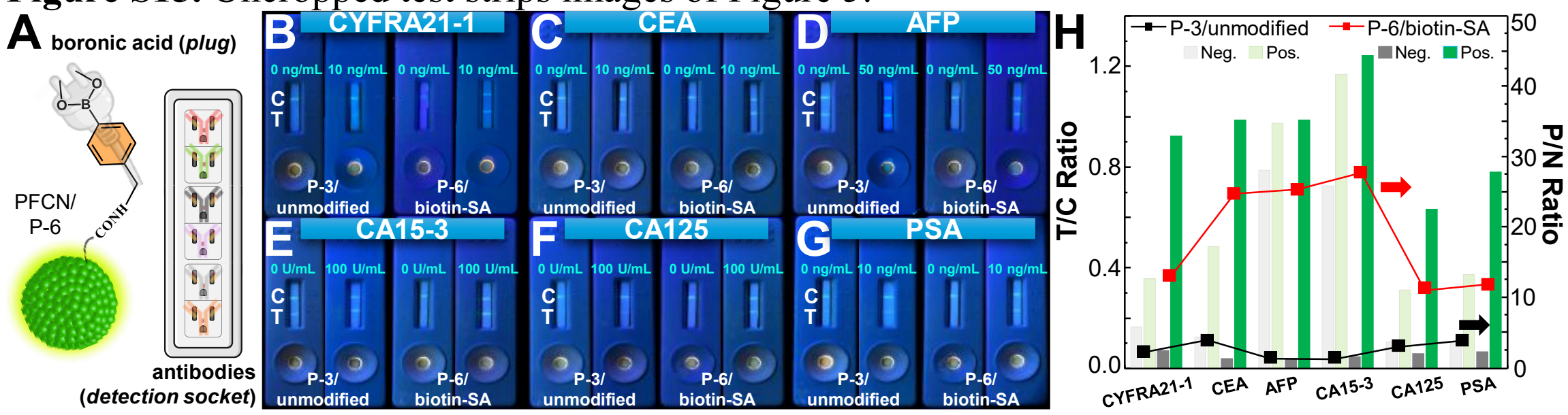

Figure S16. Uncropped test strip images of Figure 6.

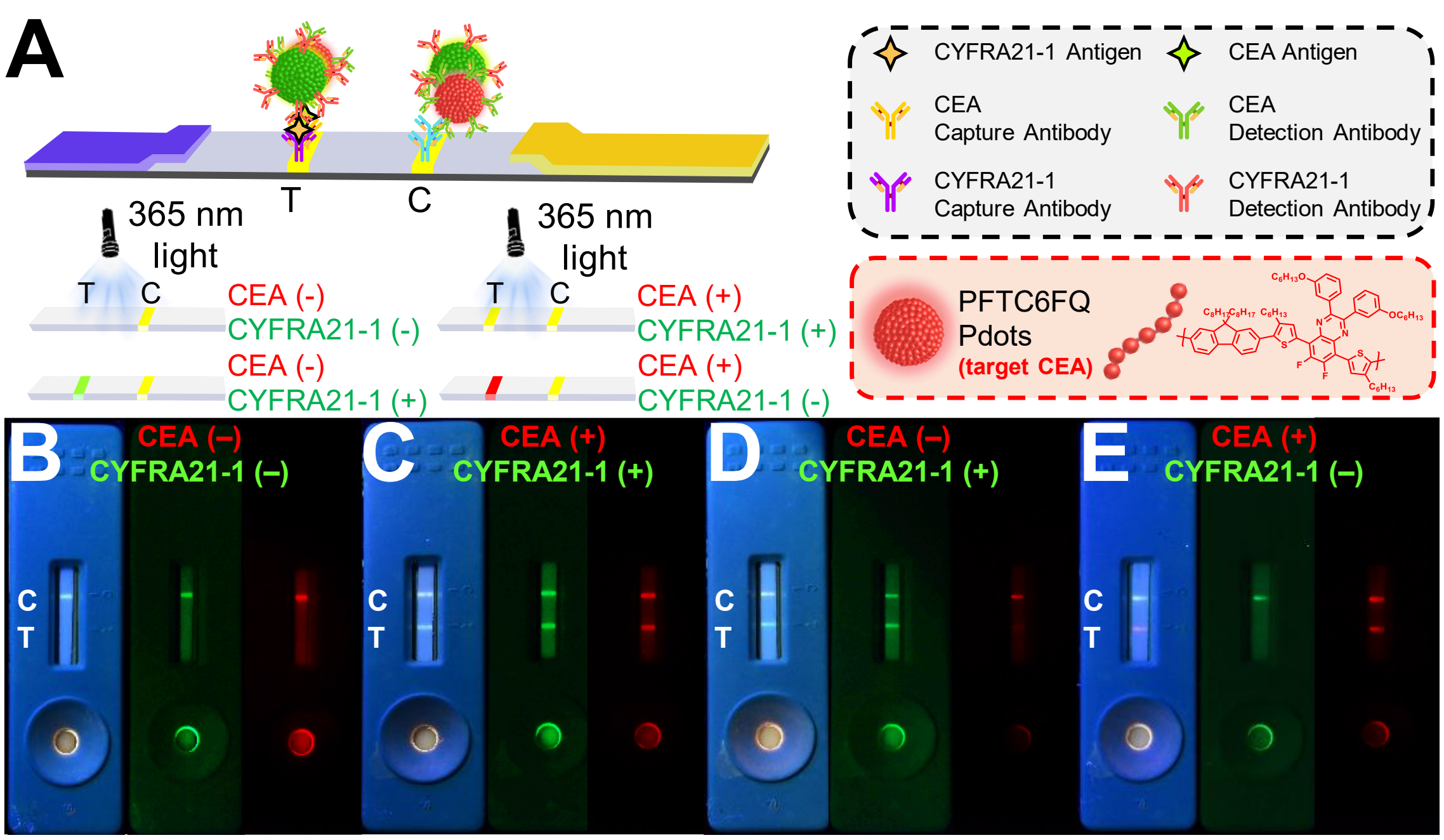

Figure S17. Uncropped test strips images of Figure 7.

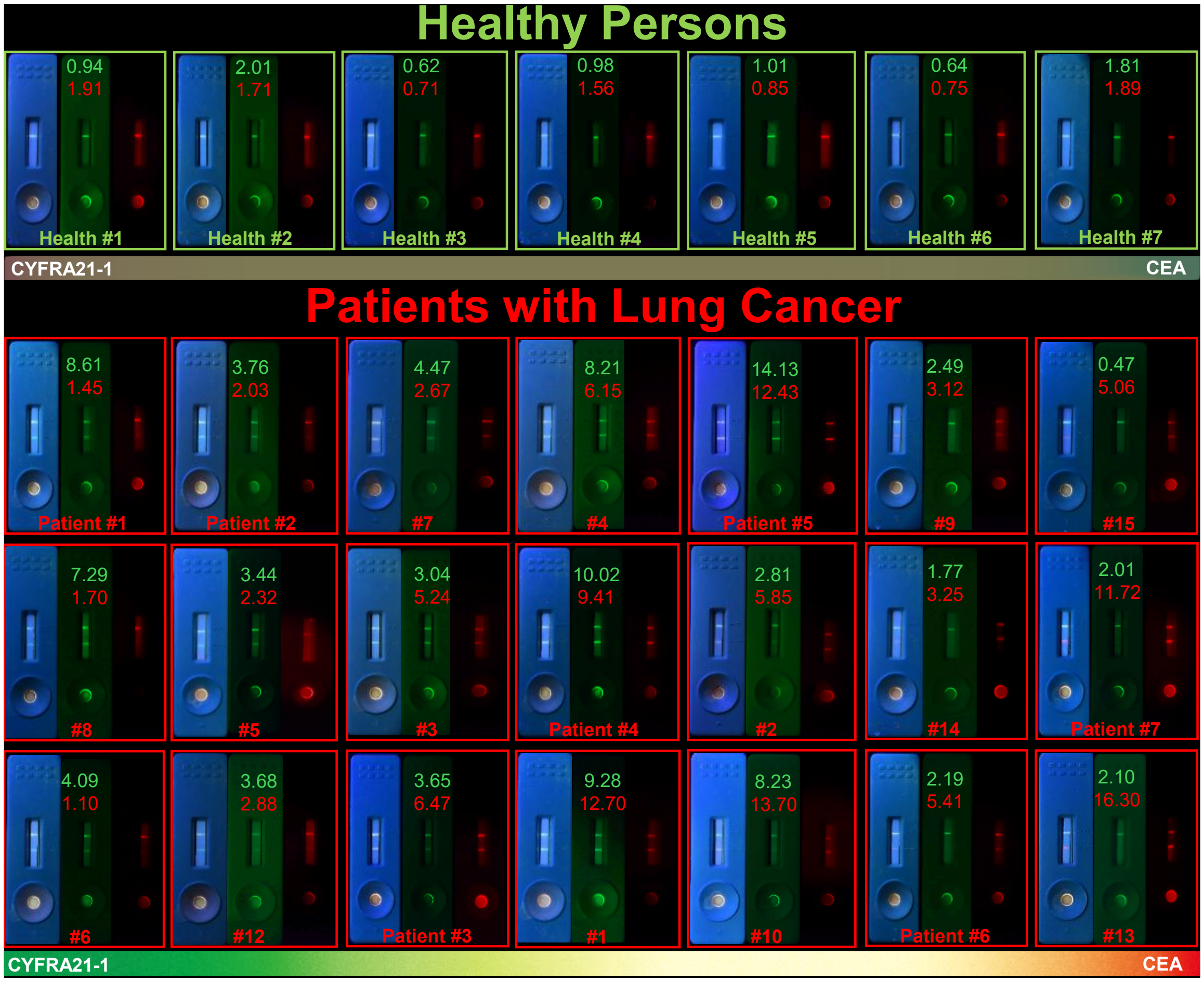

Figure S18. Uncropped test strips images of Figure 8.

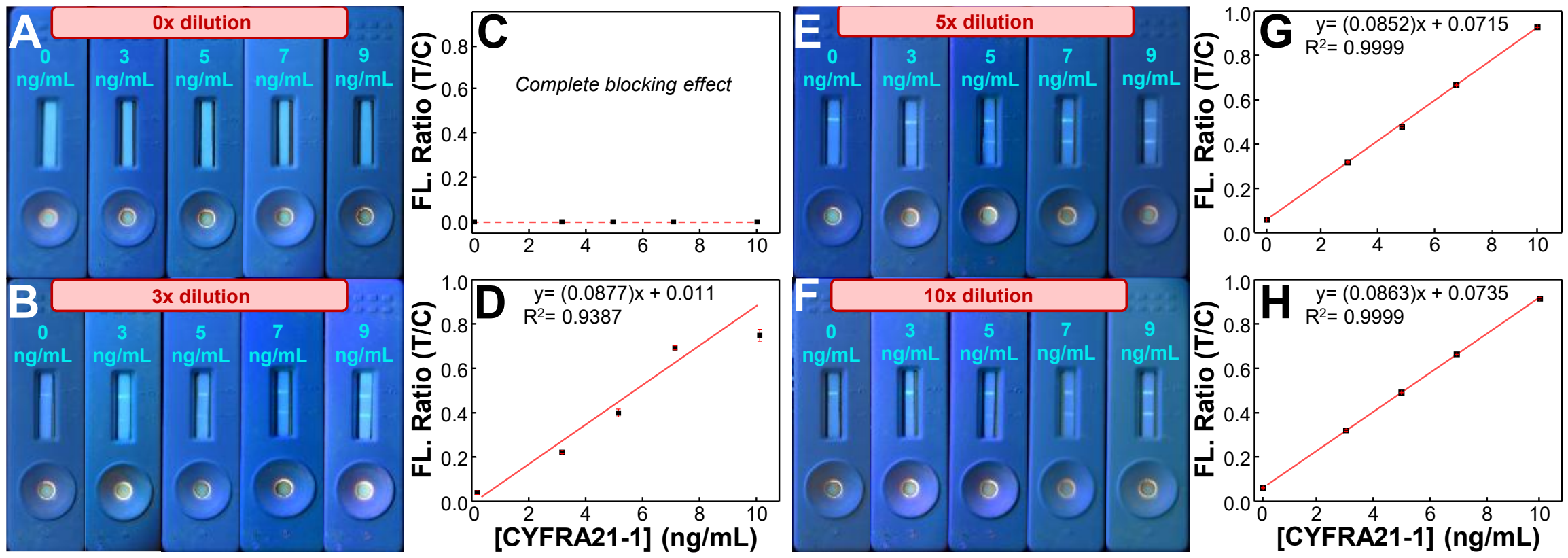

Figure S19. Matrix-matched calibration of CYFRA21-1 in pooled human serum. The P-6 and biotin-SA configuration was used throughout. (A) Strip images in undiluted serum at 0, 3, 5, 7, and 9 ng/mL CYFRA21-1. (B) Strip images at a threefold dilution. (C) T/C ratios in undiluted serum, showing complete signal suppression at every concentration. (D) Calibration at three-fold dilution, with a slope of 0.0877 and an R^2 of 0.9387. (E, F) Strip images at fivefold and tenfold dilutions. (G) Calibration at five-fold dilution, with a slope of 0.0852 and an R^2 of 0.9999. (H) Calibration at ten-fold dilution, with a slope of 0.0863 and an R^2 of 0.9999. The calibration in the running buffer alone has a slope of 0.08521. Slope recovery is therefore 99.99 percent at a five-fold dilution. Data are mean ± standard deviation of n = 3 independent measurements.

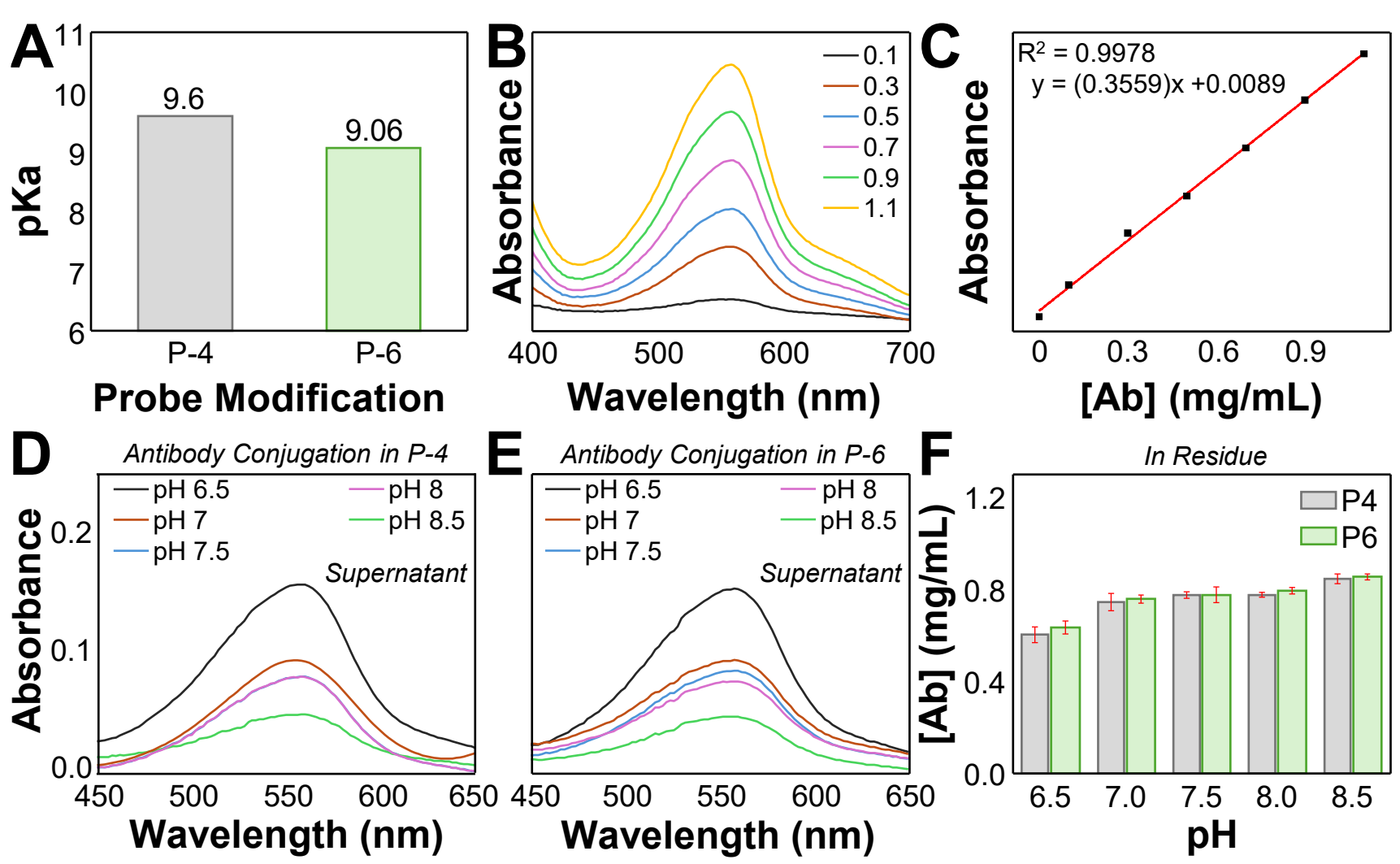

Figure S20. Computed pKa values and pH-dependent antibody loading for the two boronic acid probes. (A) Boronic acid pKa values of 9.60 for the P-4 linker and 9.06 for the P-6 linker. These came from an isodesmic proton exchange cycle referenced to phenylboronic acid. An experimental pKa of 8.8 was used for that reference acid. (B) Absorbance spectra of the BCA calibration series for anti-CYFRA21-1 from 0.1 to 1.1 mg/mL. (C) The corresponding calibration curve has an R2 of 0.9978. (D, E) Absorbance spectra of the post-conjugation supernatants for P-4 and P-6. Spectra were recorded at pH 6.5, 7.0, 7.5, 8.0, and 8.5. (F) Antibody concentration immobilized on the probe, obtained by difference. Loading rises from about 0.60 mg/mL at pH 6.5 to about 0.85 mg/mL at pH 8.5. The two linkers differ by at most 0.030 mg/mL across all tested pH values. Data are the mean ± standard deviation from n = 3 independent conjugations. Antibody was offered at 1.00 mg/mL in every case.

Table S1. Comparison of the analytical performance with previously reported antibody orientation strategies for CYFRA21-1 and CEA detection.

Orientation Strategy	Target	Linear Range (ng/mL)	LoD before Enhancement (ng/mL)	LoD after Enhancement (ng/mL)	Overall Enhancement Factor	Ref.
**Probe modifications**						
Biotin-SA (MNPs[a])	CEA	0.25-1000	-	0.25	-	[1]
Boronic acid (4-MPBA[b])	CEA	1.25-50	10	1.25	~8x	[2]
**Test line modifications**						
Protein-G (Au NPs[c])	CEA	2-50	10	0.35	~28.6x	[3]
Biotin-SA (AuNPCs[d])	CEA	0.01-0.1	28	16	~1.75x	[4]
**Dual-site modifications**						
Probe Only modification (P-3 vs P-6)	CYFRA 21-1 /CEA	0-13 /0-20	0.6760/1.129	0.0969/0.2251	~7x/~5x	This work
Test line only modification (P-6 vs P-6/biotin-SA)			0.0969/0.2251	0.0352/0.0808	~2.75x/~2.78x	
Both probe and test line modification (P-3 vs P-6/biotin-SA)			0.6760/1.129	0.0352/0.0808	~19.2x/~14x	

[a]MNPs: magnetic nanoparticles; [b]4-MPBA: 4-mercaptophenylboronic acid; [c]AuNPs: gold nanoparticles; [d]AuNPCs: gold nanopinocones.

References:

1. F. Liu, H. Zhang, Z. Wu; et al., "Highly sensitive and selective lateral flow immunoassay based on magnetic nanoparticles for quantitative detection of carcinoembryonic antigen," *Talanta* 161, no. (2016): 205-210, https://doi.org/10.1016/j.talanta.2016.08.048.

2. P. Wu, J. Song, W. Zuo; et al., "A universal boronate affinity capture-antibody-independent lateral flow immunoassay for point-of-care glycoprotein detection," *Talanta* 265, no. (2023): 124927, https://doi.org/10.1016/j.talanta.2023.124927.

3. T. Mahmoudi, B. Shirdel, B. Mansoori; et al., "Dual sensitivity enhancement in gold nanoparticle-based lateral flow immunoassay for visual detection of carcinoembryonic antigen," *Analytical Science Advances* 1, no. 3 (2020): 161-172, https://doi.org/10.1002/ansa.202000023.

4. J. Fox, D. V. B. Batchelor, H. Roberts; et al., "Gold Nanotapes and Nanopinecones in a Quantitative Lateral Flow Assay for the Cancer Biomarker Carcinoembryonic Antigen," *ACS Applied Nano Materials* 6, no. 19 (2023): 17769-17777, https://doi.org/10.1021/acsanm.3c03053.
